# A novel method for visualizing and tracking endogenous mRNA in a specific cell population in pathological neovascularization

**DOI:** 10.1038/s41598-021-81367-5

**Published:** 2021-01-28

**Authors:** Md Imam Uddin, Tyler C. Kilburn, Sara Z. Jamal, Craig L. Duvall, John S. Penn

**Affiliations:** 1 Department of Ophthalmology and Visual Sciences, Vanderbilt University School of Medicine, AA1324 Medical Center North, Nashville, TN 37232 USA; 2 Department of Biomedical Engineering, Vanderbilt University School of Engineering, Nashville, TN USA; 3 Department of Cell and Developmental Biology, Vanderbilt University School of Medicine, Nashville, TN USA; 4 Department of Molecular Physiology and Biophysics, Vanderbilt University School of Medicine, Nashville, TN USA

**Keywords:** Chemical biology, Genetics, Immunology

## Abstract

Diabetic retinopathy, retinopathy of prematurity and retinal vein occlusion are potentially blinding conditions largely due to their respective neovascular components. The development of real-time in vivo molecular imaging methods, to assess levels of retinal neovascularization (NV), would greatly benefit patients afflicted with these conditions. mRNA hybridization techniques offer a potential method to image retinal NV. The success of these techniques hinges on the selection of a target mRNA whose tissue levels and spatial expression patterns correlate closely with disease burden. Using a model of oxygen-induced retinopathy (OIR), we previously observed dramatic increases in retinal endoglin that localized to neovascular structures (NV), directly correlating with levels of neovascular pathology. Based on these findings, we have investigated Endoglin mRNA as a potential marker for imaging retinal NV in OIR mice. Also of critical importance, is the application of innovative technologies capable of detecting mRNAs in living systems with high sensitivity and specificity. To detect and visualize endoglin mRNA in OIR mice, we have designed and synthesized a novel imaging probe composed of short-hairpin anti-sense (AS) endoglin RNA coupled to a fluorophore and black hole quencher (AS-Eng shRNA). This assembly allows highly sensitive fluorescence emission upon hybridization of the AS-Eng shRNA to cellular endoglin mRNA. The AS-Eng shRNA is further conjugated to a diacyl-lipid (AS-Eng shRNA–lipid referred to as probe). The lipid moiety binds to serum albumin facilitating enhanced systemic circulation of the probe. OIR mice received intraperitoneal injections of AS-Eng shRNA–lipid. Ex vivo imaging of their retinas revealed specific endoglin mRNA dependent fluorescence superimposed on neovascular structures. Room air mice receiving AS-Eng shRNA–lipid and OIR mice receiving a non-sense control probe showed little fluorescence activity. In addition, we found that cells in neovascular lesions labelled with endoglin mRNA dependent fluorescence, co-labelled with the macrophage/microglia-associated marker IBA1. Others have shown that cells expressing macrophage/microglia markers associate with retinal neovascular structures in proportion to disease burden. Hence we propose that our probe may be used to image and to estimate the levels of retinal neovascular disease in real-time in living systems.

## Introduction

Diabetic retinopathy (DR) is a vision-threatening condition that affects a large number of diabetic patients within the working age population worldwide^1,2^. The early stage, referred to as non-proliferative DR (NPDR), is partially characterized by retinal vaso-regression (ischemia) leading to hypoxia. Proliferative DR (PDR), constitutes the late stage, and it is defined by the development of pre-retinal neovascularization characterized by the formation of neovascular structures at the vitreoretinal interface. These structures or ‘neovascular tufts’ are often associated with hemorrhaging and tractional retinal detachment that may lead to blindness. Although the pathogenic mechanisms underlying PDR are largely unknown, ischemia-induced hypoxia and the release of hypoxia-dependent vascular endothelial growth factor (VEGF), in addition to other vasoactive and/or proinflammatory factors, are of central importance^3^.

Evidence shows that circulating bone marrow derived cells (BMC) migrate to the retina in response to neovascularization^4–7^. However, the exact role of these migrating cells to induce or promote NPDR or PDR is largely unknown^8^. Additionally, retinal macrophages are associated with retinal neovascularization occurring in ischemic retinopathies, releasing pro-angiogenic and pro-inflammatory mediators possibly contributing to the neovascular response^9^. However, technical difficulties are the major hurdle against characterizing this small number of activated cells in the retina. In addition, contributions from other cells to retinopathy is a possibility.

Endoglin (CD105) is a transmembrane auxiliary receptor for transforming growth factor-beta (TGF-β) that is predominantly expressed in proliferating vascular endothelial cells^10–12^, and bone marrow-derived endothelial progenitor cells^13^. Very little is known about the role of endoglin in human PDR, though soluble endoglin (sEng) levels are increased in the vitreous^14^ and blood^15^ of PDR patients and in the retinas of experimental models of diabetes^16^. It is speculated that sEng is a proteolytic cleavage product of the full-length protein^17^. The shRNA knockdown and use of neutralizing antibodies against endoglin in cell-based assays that model angiogenic components of PDR, suggested that endoglin has proangiogenic function^18^. In a previous study, we observed that endoglin (CD105) protein is associated with neovascularization^19^. In line with these observations, real-time imaging of endoglin mRNA that correlates with the onset, progression and resolution of neovascularization could possibly predict, and could serve as a useful imaging tool. However, molecular imaging of specific mRNAs in living retina remains a major challenge. Fluorescence in situ hybridization (FISH) is a powerful method to visualize intracellular mRNA localization in ex vivo tissue preparations and is capable of distinguishing RNA molecules that differ in only a single base^20^. Other hybridization methods include the use of molecular beacon^21^ and forced intercalation probes (FIT)^22^. Additional methods to visualize mRNA include covalent modification of mRNA^23^, mRNA binding proteins, and reporter protein expression by trans-splicing to visualize mRNA. However, most of these hybridization methods require the use of fixed tissues or endogenously labelled target mRNA for imaging and tracking. Recent development of gold-mediated targeted delivery of oligonucleotides facilitates the real-time imaging of mRNA in living cells^24^. In this current study, we have designed and synthesized AS-Eng shRNA–lipid conjugates for targeted imaging of endoglin mRNA that is associated with neovascularization in living retinas without using any toxic transfection reagents. We consider this an important step in the translation of mRNA imaging to the clinic to monitor disease onset, pathologic progression and response to therapy.

## Results

### Design and synthesis of shRNA–lipid conjugates

**Figure 1:**
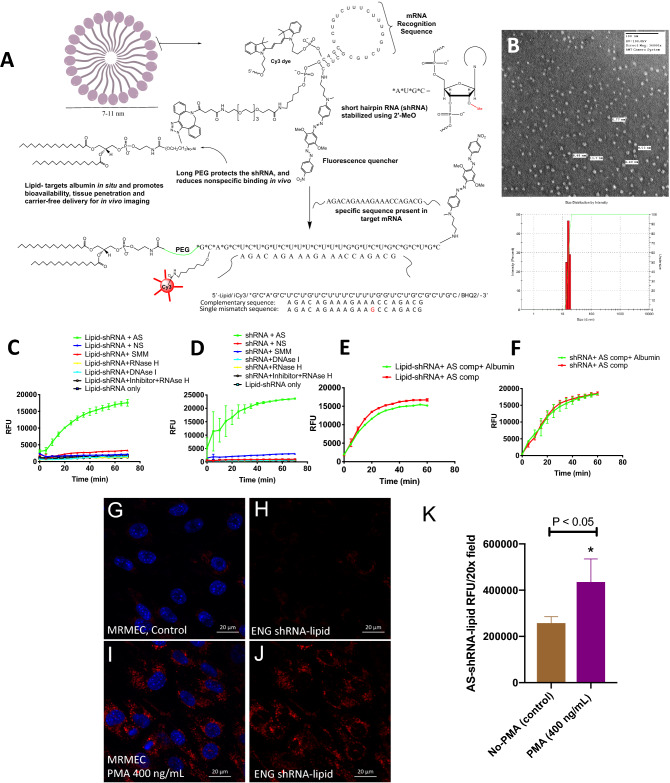
Design and characterization of AS-Eng shRNA–lipid. (**A**) Schematic drawing and hybridization motif of shRNA–lipid conjugates showing the design incorporating anti-sense sequence complementary to the endoglin mRNA. (**B**) Transmission electron microscopy (TEM) images and dynamic light scattering (DLS) measurement of the synthesized shRNA–lipid conjugates. (**C**,**D**) Both shRNA and shRNA–lipid conjugates are highly specific for their complementary sequences and do not hybridize with a single-mismatched (SMM) sequence that is otherwise complimentary. (**E**,**F**) Lipid-shRNA hybridization reactions to an exogenous complementary sequence in the presence and absence of albumin. The resulting increased fluorescence observed both conditions indicate albumin does not effect hybridization to the target sequence. (**G**–**K**) Localization of AS-Eng AS-shRNA–lipid in mouse retinal microvascular endothelial cells (MRMECs) using fluorescence microscopy. ENG mRNA is induced in MRMECs by treating the cells with 400 ng/mL phorbol 12-myrestyle 13-acetate (PMA) for 24 h. DAPI is used to counterstain the nucleus (blue). Fluorescence intensities were measured computationally using ImageJ software (n = 6). Statistical significance *P < 0.05. *AS *anti-sense complementary oligonucleotide, *NS *non-sense oligonucleotide, *SMM *single-mismatched oligonucleotide.

In order for the probe design, we used computational analysis of the shRNA to target endoglin mRNA with high specificity. The region in endoglin mRNA was selected on the basis of accessibility of shRNA as predicted by RNA secondary structure predictions using MFOLD software^25^. Then, the best candidate sequence was determined using the OLIGOWALK software^26^ based on the probe sequence predicted to bind most stably to its complementary sequence. After designing and selecting the best sequence, nuclease-resistant shRNA were synthesized with 2′-*O*-methylribonucleotide (2′-OMe) modified RNA chemistry. A fluorescence dye introduced at the 5′-end was quenched by black-hole quencher-2 (BHQ2) introduced at the 3′-end of the oligonucleotide. The AS (or NS)-shRNA products were purified using high-pressure liquid chromatography through a C-18 reverse-phase column. A diacyl-lipid was then conjugated in two steps using our previously developed method as described^27^ in order for transfection agent-free delivery of shRNA to the neovascular lesions. Physical properties of shRNA–lipid conjugates were monitored using transmission electron microscopy (TEM) and dynamic light scattering (DLS) (Fig. 1). Since the shRNA–lipid conjugates contain a hydrophilic and lipophilic components, it is likely that they could form lipid–micelle structures. From our DLS measurements we observed that in solution shRNA–lipid conjugates contain one major population of nanoparticles by volume, suggesting good measurement quality. However, polydispersity index value of 0.203 demonstrates a broad size range within the population suggesting the presence of multiple species/nanoparticles. This multi-species nanoformulation may be due to shRNA–lipid conjugates with a series of PEG lengths as observed in ESI-TOF MS data of the shRNA–lipid conjugates at around 15 kDa as shown in Figure S1, contributing to high polydispersity index.

Signal-to-background ratios were measured from hybridization kinetics in the presence of the target sequence (Fig. 1)^28^. Upon hybridization to a complementary oligonucleotide sequence present in the target mRNA, the fluorophore is de-quenched, allowing strong fluorescence emission by a factor of several thousand with sensitivity enhancement of about 100-fold as shown in Fig. 1C,D and Figure S3. The target sequence detection is highly specific and demonstrated the capacity to discriminate a single mismatch in the target mRNA. A non-sense probe (NS-shRNA–lipid) proved virtually unresponsive. To test the nuclease sensitivity, AS-Eng shRNA–lipid conjugates were treated with DNase I and RNase H and subsequent changes in fluorescence were measured as function of time as shown in Fig. 1C,D. Modification with 2′-*O*-methylribonucleotides for deoxyribonucleotides within the backbone renders the AS-Eng shRNA–lipid resistant to cleavage by DNase I and their RNA became refractory to digestion by RNase H, consistent with previously reported results^29^. Analysis of shRNA–lipid binding kinetics with complementary sequence in presence or absence of albumin showed that the shRNA–lipid bound with complementary mRNA recognition sequences regardless of the presence of albumin (Fig. 1E,F). In addition, we have used AS-shRNA–lipid for imaging Endoglin mRNA expression in mouse primary retinal microvascular endothelial cells (MRMEC). ENG mRNA was induced in MRMECs by treating the cells with 400 ng/mL phorbol 12-myrestyle 13-acetate (PMA) for 24 h. AS-shRNA–lipid derived fluorescence was minimally observed in untreated control MRMECs and the fluorescence significantly increased in PMA treated MRMECs, supporting the expression of ENG mRNA in PMA treated cells (Fig. 1G–K). Thus, AS-shRNA–lipid could be used for imaging endogenous mRNA in cultured retinal cell and potentially other cell types.

### Distribution of endoglin (ENG) mRNA in OIR retina

**Figure 2:**
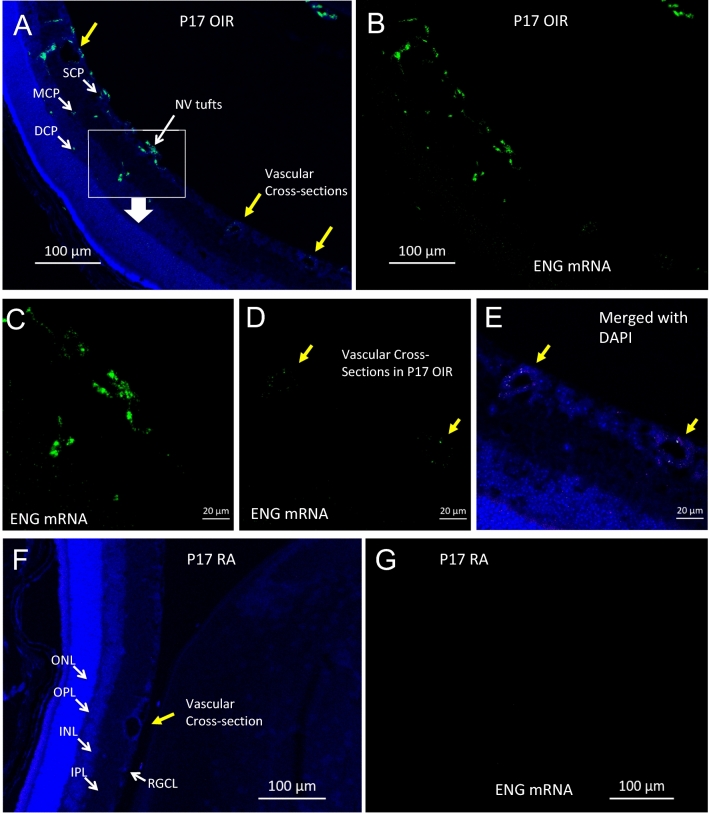
Fluorescence in situ hybridization (FISH) imaging to visualize endoglin (ENG) mRNA expression in transverse retinal sections from P17 OIR and P17 RA control mice. An intense green punctate, endoglin mRNA-dependent fluorescence is observed in OIR retinal cross section in cells around the vascular structures including superficial-, middle- and deep-capillary plexuses as shown in (**A**,**B**). DAPI staining was performed to identify the retinal layers. Scale bar 100 μm. (**C**) magnification view of the neovascularization (NV) in (**A**). (**D**,**E**) magnification view of the vascular cross-section (yellow arrows) shows minimal fluorescence in mature endothelial cells. Minimal fluorescence was observed in RA control retinas at P17 as shown in (**F**,**G**). *SCP *superficial capillary plexus, *MCP *middle capillary plexus, *DCP *deep capillary plexus), *RGCL *retinal ganglion cell layer, *IPL *inner plexiform layer, *INL *inner nuclear layer, *OPL *outer plexiform layer, *ONL *outer nuclear layer. Scale bar 20 μm.

We also used fluorescence in situ hybridization (FISH) to localize expression of endoglin mRNA in the excised OIR retina as shown in Fig. 2, and immunostaining was used to co-localize endoglin (CD105) in F4/80-positive cells (Figure S7). Retinal tissues were harvested from OIR and RA controls at P17 for ex vivo analysis of endoglin mRNA distribution using confocal microscopy. With this technique, we were able to localize endoglin mRNA in neovascular tufts presumably in endothelial cells and also in microglial/macrophages in the retina. In addition, we observed that endoglin mRNA was localized around the capillaries within the OIR retina. However, fluorescence was not detectable in age-matched healthy control retinal cross sections as shown in Fig. 2F,G.

### Direct imaging of endogenous mRNA in living retina using AS-Eng shRNA–lipid

**Figure 3:**
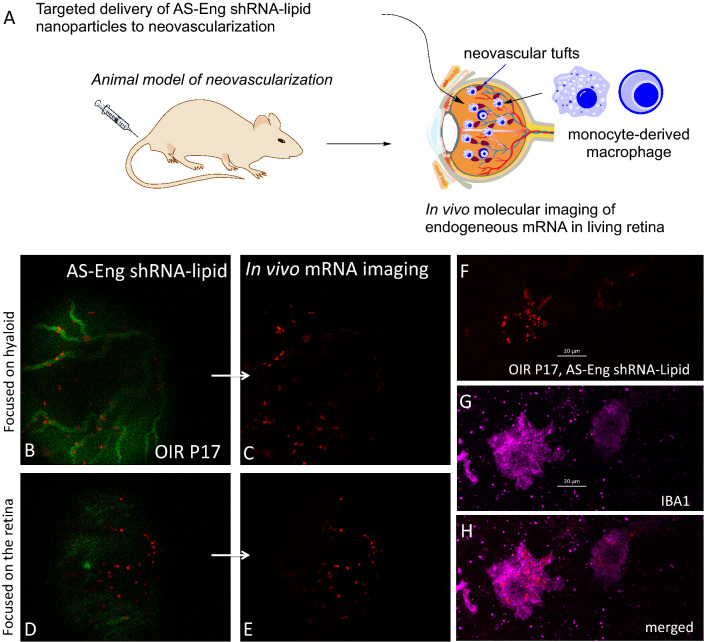
Live imaging of endogenous mRNA in the OIR retina from a P17 OIR mouse receiving an intraperitoneal injection of AS-Eng shRNA–lipid (probe). Schematic drawing representing in vivo probe delivery to neovascular tufts (**A**). Fluorescein angiography was performed to visualize the hyaloid (**B**) and the superficial (**C**) vasculatures. An intense endoglin mRNA-dependent red punctate fluorescence resulting from internalization of the AS-End shRNA–lipid by cells associated with the hyaloid vasculature (**B**,**C**) and pre-retinal neovascular tufts (**D**,**E**). Ex vivo imaging of flatmounted retinas previously imaged in vivo (**F**–**H**). IBA1 is a microglia/macrophage marker. An intense endoglin mRNA-dependent red punctate fluorescence localized to IBA-1 positive microglial cells as observed in in vivo images (**F**–**H**). Retinal flatmounts were immunostained with antibodies against IBA1.

We used our imaging probe, the AS-Eng shRNA–lipid conjugate that incorporates anti-sense sequence complementary to endoglin mRNA for molecular imaging of neovascularization in the OIR retina (Fig. 3)^30^. After intraperitoneal injection in OIR animals, we observed that AS-Eng shRNA–lipid yielded a strong punctate fluorescence in cells that were also positive for ionized calcium-binding adaptor molecule 1 (IBA1), presumably due to hybridization with endoglin mRNA in these cells (Fig. 3F–H). In addition, we observed that endoglin (CD105) is also associated with F4/80-positive cells in P17 OIR retinas (Figure S7). NS-shRNA–lipid conjugate showed minimal fluorescence in the P17 OIR retina as shown in Figure S2 and Figure S5.

**Figure 4:**
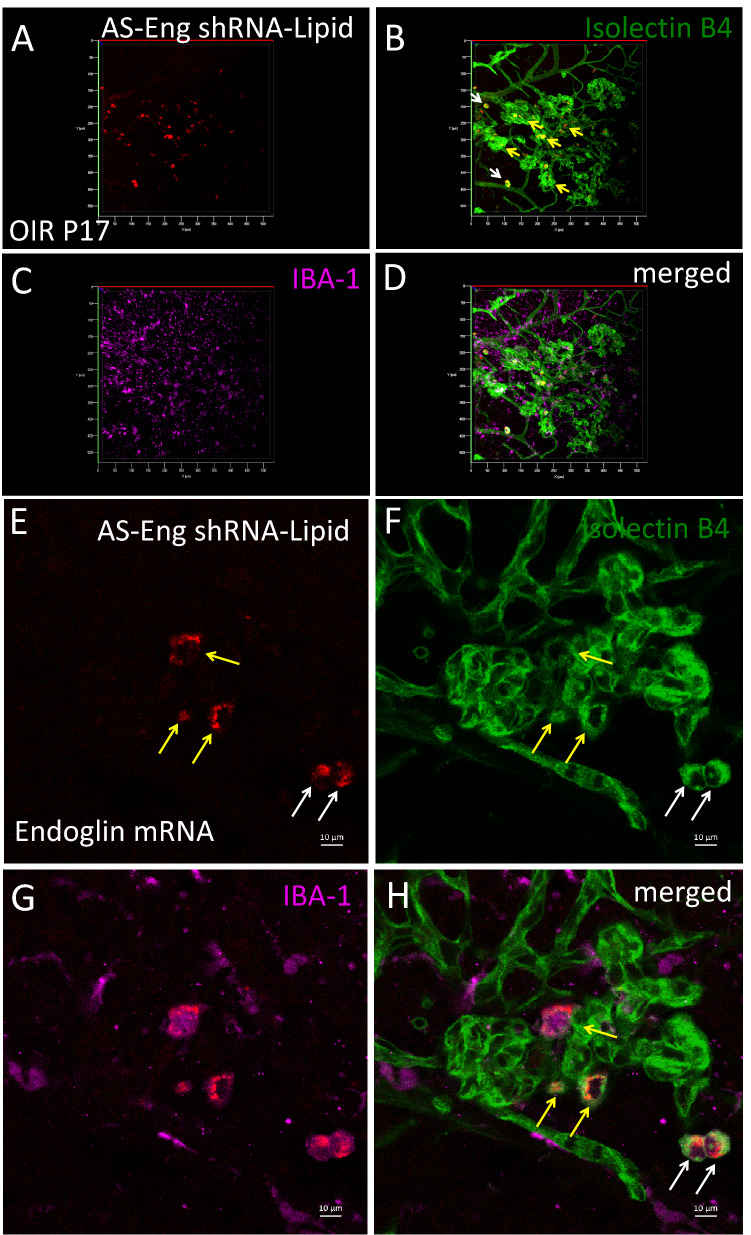
Ex vivo validation of AS-Eng shRNA–lipid fluorescence localized with IBA1 positive cells that are associated with neovascular tufts in mouse P17 OIR retina. Isolectin B4 was used to visualize the retinal vasculatures. (**A**–**D**) Showing shRNA–lipids are *associated with neovascularization* and not in normal vasculatures. The OIR mice received intraperitonel injections of AS-Eng shRNA–lipid conjugates. Eighteen hours post-injection, retinal tissues were analyzed ex vivo. AS-Eng shRNA–lipid fluorescence was localized in IBA1 positive cells (arrows), suggesting that endolgin and IBA1 positive activated microglia/macrophages are associated with neovascularization^31^. (**E**–**H**) Showing shRNA–lipids are *associated with IBA1 positive cells* in neovascularization in the superficial capillary plexus. Strong fluorescence emission presumably due to hybridization with endoglin mRNA in these IBA1 positive cells localized around neovascularization, showing the probe delivery to the neovascular tufts. Minimal fluorescence was observed in the normal endothelial cells that are also positive for endoglin mRNA, suggesting that the probe hybridization might occur at the site away from these microvascular endothelial cells and migrated to the site of neovascularization.

**Figure 5:**
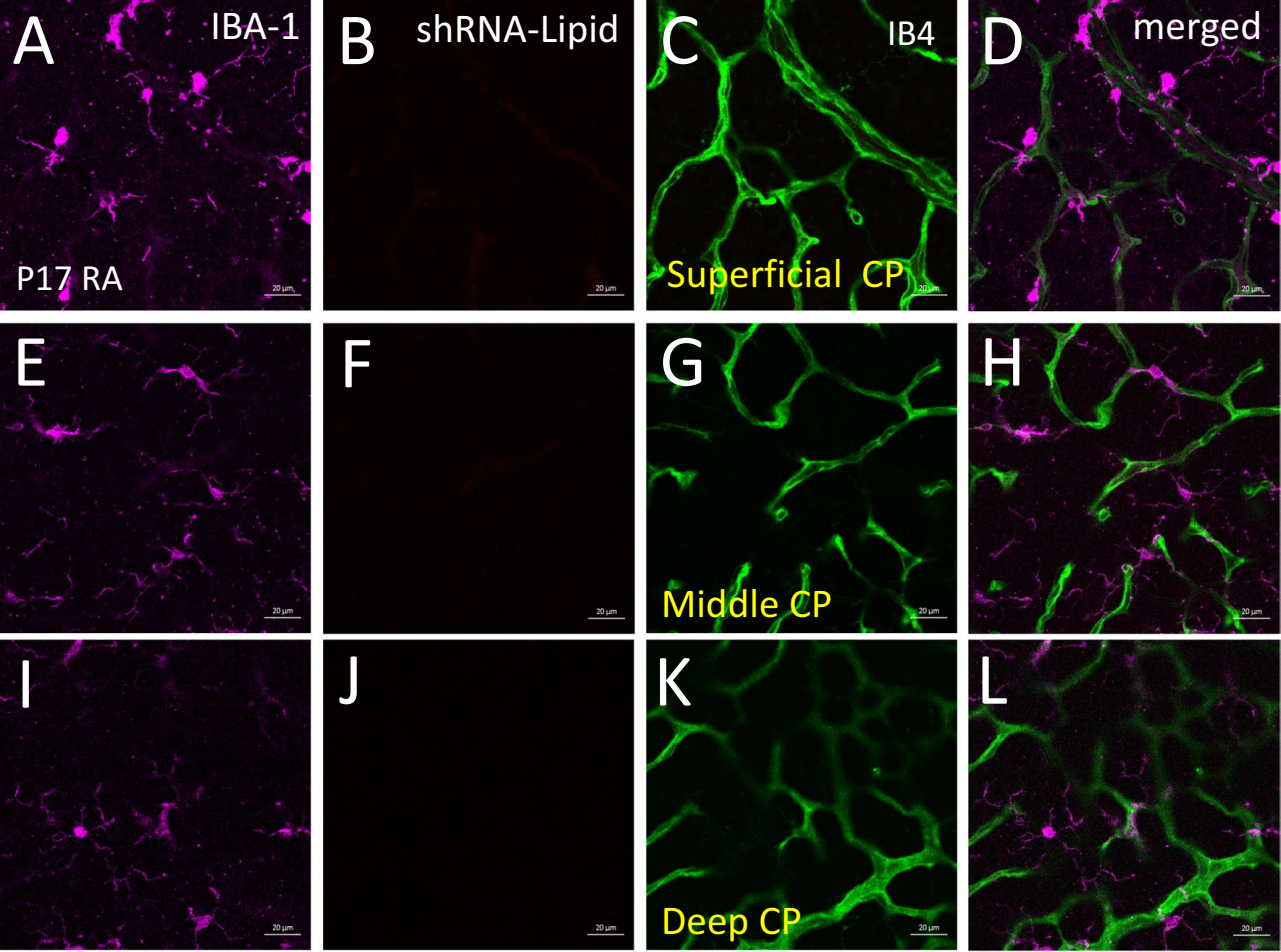
Ex vivo flat mounts of P17 mice maintained in normoxia and receiving intraperitoneal injections of AS-Eng-shRNA–lipid conjugates (normoxic controls). Flatmounts were immunostained with antibodies against IBA1 (**A**,**E**,**I**). IBA1 is a microglia/macrophage marker. Isolectin B4 was used to visualize the superficial capillary (SCP), middle capillary (MCP) and deep capillary (DCP) plexuses (**C**,**G**,**K**). Only minimal background AS-Eng shRNA–lipid-dependent fluorescence was observed, and not detected in SCP (**B**), MCP (**F**) and DCP (**J**). IBA1 positive cells were observed juxtapositioned to SCP (**A**), MCP (**E**) and DCP (**I**) and appeared to be ramnified. These data suggest IBA1 positive cells do not yield an AS-Eng-shRNA–lipid-dependent fluorescence in age-matched normal control mice and also suggest that the shRNA–lipids have no effect on retinal microglial activation. Scale bar 20 µm.

**Figure 6:**
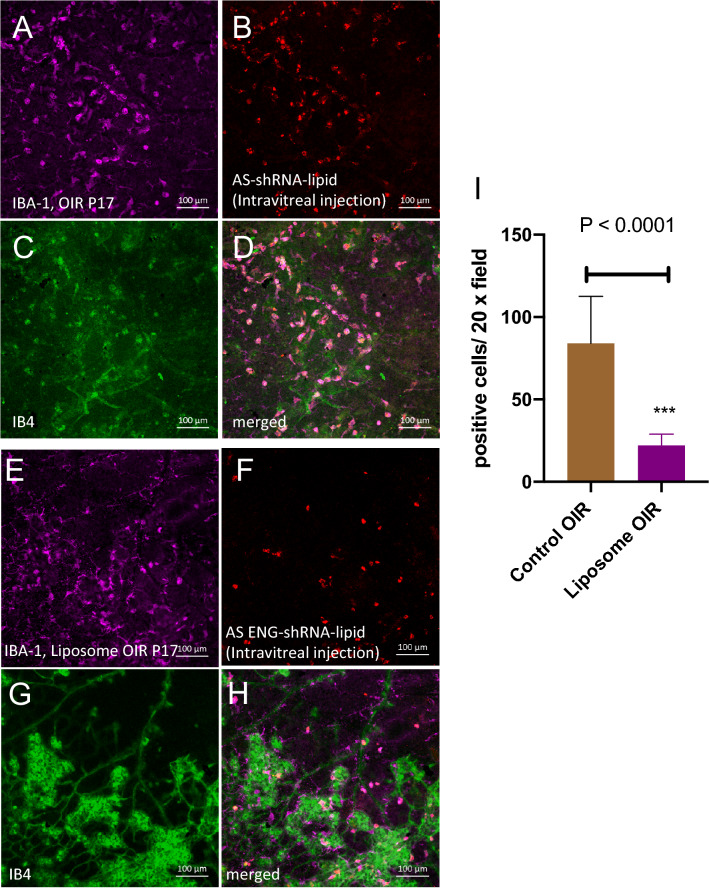
Depletion of microglia/macrophages using clodronate-liposome in mouse OIR reduces the AS-Eng shRNA–lipid derived fluorescence in the retinas compared to control PBS-injected OIR retinas. AS-Eng shRNA–lipid derived fluorescence was monitored in mice with intraocular injection of AS-Eng shRNA–lipid. IBA1 was used to visualize the microglia/macrophages in the retina and IB4 was used to visualize the vasculatures. (**A**–**D**) A large number of IBA1 positive microglial/macrophages were observed in the control PBS-liposome injected OIR retinas that were also positive for AS-Eng shRNA–lipid derived fluorescence. (**E**–**H**) However, number of AS-Eng shRNA–lipid positive cells was decreased significantly in the clodronate-liposome injected retinas (**B** vs **F**) as shown in l (n = 9 each sample group). Scale bar 100 µm.

**Figure 7:**
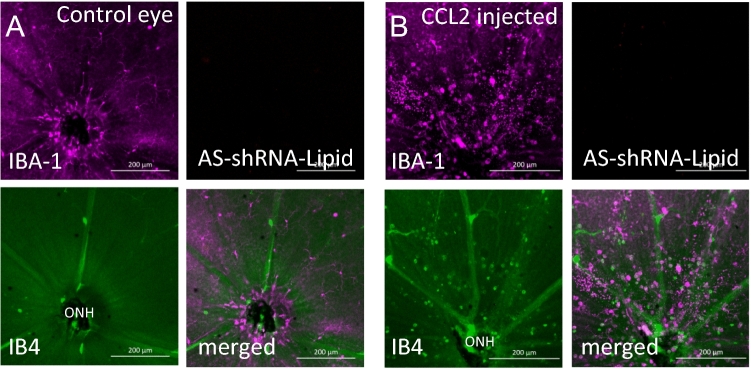
Endoglin mRNA targeted AS-shRNA–lipid derived fluorescence was monitored in mice with intraocular injection of recombinant CCL2 protein in one eye and used the other eye as un-injected control to confirm the specificity of the AS-shRNA–lipid probes. IBA1 was used to visualize the microglia/macrophages in the retina and IB4 was used to visualize the vascular structures. Under anesthesia, CCL2 (2 μL) was injected intraocularly followed by intraperitoneal injection of AS-shRNA–lipid (0.5 mg/kg). After 18 h mice were sacrificed for analysis (n = 5). (**A**) Ramified IBA1 positive microglia/macrophages were observed in the un-injected control eye. (**B**) A large number of IBA1 positive amoeboid shaped microglia/macrophages were observed in the CCL2 recombinant protein injected eyes, generally around the optic nerve head (ONH). However, AS-shRNA–lipid derived fluorescence were not observed in IBA1 positive cells in un-injected control eyes as well as in CCL2 injected eyes, suggesting that the AS-shRNA–lipids are specific for microglia/macrophages that are associated with neovascularization.

**Figure 8:**
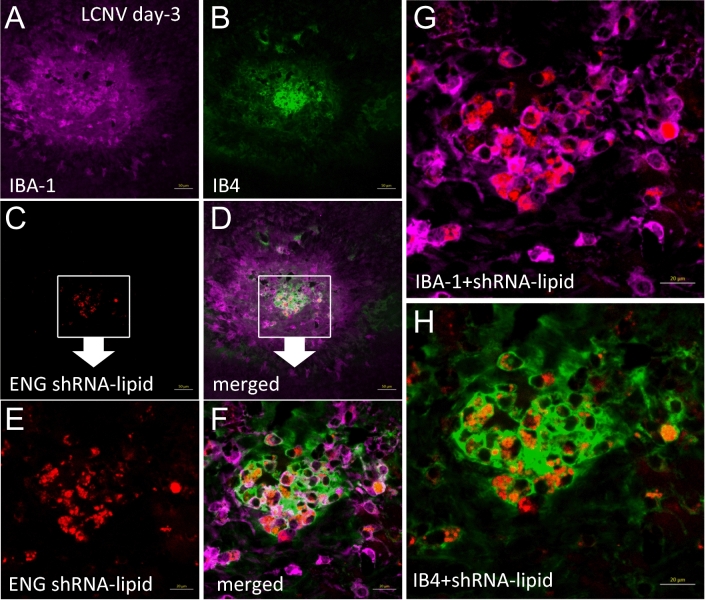
Co-localization of AS-Eng shRNA–lipid fluorescence with IBA1 positive cells in a mouse model of laser-induced choroidal neovascularization (LCNV). The LCNV mice received intraperitonel injections of AS-Eng shRNA–lipid conjugates on day-3 post-laser injury. Eighteen hours post-injection, RPE-choroid complex tissues were analyzed ex vivo. (**A**,**B**) IBA1 was used to visualize the microglia/macrophages and IB4 was used to visualize the neovascular lesions. (**C**,**D**) Showing AS-Eng shRNA–lipids are *associated with IBA1* + *microglia/macrophages in choroidal neovascularization*. (**E**,**F**) magnification of (**C**) and (**D**) images respectively. AS-Eng shRNA–lipid fluorescence is localized in IBA1 positive cells, suggesting that endolgin positive microglia/macrophages are associated with choroidal neovascularization. (**G**,**H**) Larger view of the LCNV lesion showing co-localization of AS-Eng shRNA–lipids with IBA1 positive macrophages, as shown in (**G**). Interestingly, some of the AS-Eng shRNA–lipids positive IBA1 stained cells are also stained positive for IB4, as shown in (**H**).

To use this method as a tool for predicting neovascularization in proliferative retinopathy, we analyzed the excised retina and the results are shown in Fig. 4. Strong fluorescence (red punctate) was observed in cells that were also associated with neovascular tufts identified by structure and Isolectin B4 counterstaining. We observed two morphologically distinct cell populations positive for AS-Eng shRNA–lipid-derived fluorescence that were also positive for IBA1. IBA1 is a marker for retinal microglia and macrophages^7^. We did not observe AS-Eng shRNA–lipid derived fluorescence in ramified IBA1-positive cells that resided around the deep capillary plexus of the OIR retina. Interestingly, AS-Eng shRNA–lipid-derived punctate fluorescence was observed in perinuclear regions of the non-ramified IBA1-positive cells and throughout the cytoplasm. Notably, we did not observe AS-Eng shRNA–lipid-derived fluorescence in the retinal microvascular endothelial cells in the neovascular tufts that are also positive for endoglin mRNA as shown in Fig. 2. This observation suggests that after intraperitoneal injections, shRNA–lipid conjugates were internalized by the circulating monocyte-derived macrophages outside the ocular tissues, as shown in Figure S6, which then migrated to the retina. This migration could be a response to neovascularization and could be used as measure for the severity of the disease progression and also treatment response in proliferative vascular diseases. To confirm the specificity of the AS-Eng shRNA–lipid conjugate, we performed the following control experiments: (1) We injected the same AS-Eng shRNA–lipid conjugate to age-matched normal healthy control animals as shown in Fig. 5. We observed that IBA1-positive cells are distributed around the vasculature in the superficial, middle and deep capillary plexus and were minimally positive for AS-Eng shRNA–lipid-derived fluorescence in all three layers in healthy control retinas (Fig. 5, see also Figure S2). (2) NS-shRNA–lipid conjugate showed minimal fluorescence in the same OIR retina as shown in Figure S5 and also in Figure S2. (3) Depletion of the circulating monocyte-derived macrophages using intraperitoneal injection of clodronate liposome reduced the AS-shRNA–lipid-dependent fluorescence in P17 OIR retina (Fig. 6). To confirm the specificity of AS-Eng shRNA–lipid for ENG positive cells that are associated with neovascularization, we administered AS-Eng shRNA–lipid to adult animals receiving intraocular injection of CCL2 (MCP1) to promote infiltration of monocyte-derived macrophages and monitor AS-Eng shRNA–lipid derived fluorescence (Fig. 7). Ex vivo analysis of the excised retinal tissues showed that AS-shRNA–lipid derived fluorescence was absent in infiltrating microglia/macrophages in CCL2 injected eyes, suggesting that the AS-shRNA–lipid are specific for microglia/macrophages that are associated with neovascularization. Furthermore, we extend the feasibility of this imaging method to other neovascularization condition, such as laser-induced choroidal neovascularization (LCNV), the results are shown in Fig. 8. We observed that CNV lesions are associated with IBA1 positive microglia/macrophages and AS-Eng shRNA–lipids are associated with these IBA1 positive cells in choroidal neovascularization supporting the applicability of this imaging technique to other neovascular disease.

### In vivo bio-distribution and toxicity of AS-Eng shRNA–lipid conjugates

**Figure 9:**
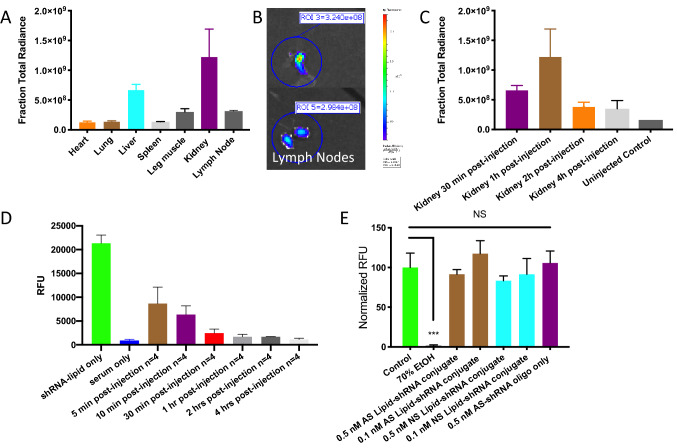
In vivo bio-distribution, pharmacokinetics and toxicity of AS-Eng shRNA–lipid conjugates. (**A**–**C**) Ex vivo imaging of the isolated organs from AS-Eng shRNA–lipid injected animals showed that these probes have prolong bioavailability in vivo for tissue uptake and are cleared through renal secretion after 4 h. The shRNA–lipid conjugates might deliver to the lymph nodes as shown in (**B**), where they might internalized into the macrophages and migrated to the retina. (**D**) Plasma concentration time profile of shRNA–lipid showing its clearance after one hour. (**E**) Cellular uptake and in vitro toxicity of AS-Eng shRNA–lipid in retinal microvascular endothelial cells were assessed by the live-dead assay using Calcein AM. Lipid-shRNA did not significantly reduce MRMEC viability at 0.1 and 0.5 nM concentrations compared to normal serum treated cells. See Figure S8 for additional data for viability assays using 0.5 µM AS-, NS-shRNA with and without lipid and dye controls. All showed no significant reduction of cell viability in presence of these compounds at 0.5 µM concentrations.

AS-Eng shRNA–lipids have prolonged bioavailability, more than an hour in vivo for tissue uptake and the unbound probes are cleared through renal secretion mostly after 4 h (Fig. 9). After intraperitoneal injections, AS-Eng shRNA–lipid conjugate clears mostly through kidney and after eighteen hours post-injection, the imaging agents were localized in IBA1 positive cells in the retina. To confirm the safety of shRNA–lipids, we used a live-dead assay to monitor cell viability after treatment with AS-Eng shRNA–lipid. Mouse retinal microvascular endothelial cells (MRMECs) were incubated with AS-Eng shRNA–lipid or NS-shRNA–lipid, and live-dead assay using Calcein AM confirmed that, independent of their nucleotide sequence, shRNA–lipids were minimally toxic to the retinal cells as shown in Fig. 9E and Figure S8. We saw no evidence of toxicity in any retinas examined after shRNA–lipid administration in vivo.

## Discussion

Regulated expression of endoglin in macrophages was demonstrated before^31^. Also, endoglin can be induced using phorbol-esters in monocytic-cells differentiated into macrophages, suggesting that inflammation might initiate phenotypic differentiation of monocytes. Tissue macrophages play a key role to promote vasculogenesis during development^32^. In addition, macrophages were observed in the retina during hyaloid degeneration and in response to neovascularization such as that occurring in proliferative diabetic retinopathy^33^. However, the exact role of macrophages in neovascularization is largely unknown. Molecular imaging of specific mRNA biomarkers in activated microglia/macrophages could uncover the role of these cells in the pathogenesis of proliferative retinopathy. Reports have shown the use of diacyl-lipid conjugated siRNA as an efficient delivery method to lymphatic system through ‘albumin hitchhiking’ where they could efficiently internalized into the phagocytes and increase the T-cell priming^30^. In the current study, we have used stabilized short hairpin RNA (shRNA) that are conjugated to diacyl-lipid (shRNA–lipid) to image endogenous mRNA in microglia/macrophages in vivo and track these cells to uncover the role of activated cells contributing to retinopathy. Overall, we hypothesized that after intraperitoneal injection, the lipid moiety of the AS-Eng shRNA–lipid conjugates protects the shRNA from degradation and blocks off-target extracellular interactions. This allows for efficient delivery of the conjugates to lymphatic system through ‘albumin hitchhiking’ where they might efficiently internalized into the phagocytes tagging endoglin mRNA and then migrated to the neovascular tufts in the OIR retina. This combined molecular feature of the lipid-conjugates offer significant advantages over other imaging methods. For example, the probe becomes fluorescent upon hybridization to target-mRNA, allowing non-invasive optical imaging in the retina. After intraperitoneal injection, the lipid conjugate is chaperoned by albumin throughout systemic circulation and is efficiently delivered to the target tissues and retained without the need for potentially toxic transfection reagents. Albumin is the most abundant serum protein (> 40 mg/mL) and has a circulation half-life of about 20 days^34^, making it a natural chaperone for systemic delivery of shRNA conjugates^35^. Our data indicate that this chaperone activity greatly facilitates the delivery of our shRNA–lipid conjugates to ocular tissues. We have also found that lipid modification of shRNA improves resistance to nucleases and enhances cellular internalization^36^. The main objective of this study is to target the altered monocyte population by imaging mRNA biomarkers and predict the ‘onset’ of neovascularization. Thus, our new imaging modality would provide a platform by targeting the altered monocyte population to predict neovascularization in proliferative retinopathy conditions, a common complication observed in proliferative diabetic retinopathy (PDR) and retinopathy of prematurity (ROP) in human. Overall, strong evidence from our data in Figs. 3, 5 and 8 suggest that AS-shRNA–lipid could be used for detecting altered monocyte-population selectively in neovascularization, which could be used as a diagnostic tool to predict the disease onset, track its progression and monitor treatment response.

In summary, we have developed a direct method for imaging specific mRNAs in living ocular tissues using diacyl-lipid-conjugates of antisense short hairpin RNA (AS-Eng shRNA–lipid). These probes are readily internalized by cells that incorporate into retinal neovascular lesions, allowing molecular imaging of vascular disease of the retina. These findings may provide a framework for a new strategy to detect and monitor retinal and choroidal neovascularization, e.g. its onset, progression and response to therapy.

## Methods

All chemicals were purchased form Sigma-Aldrich (St. Louis, MO) and used as received unless otherwise noted. The mouse primary retinal microvascular endothelial cells (MRMEC) were obtained from Cell Biologics Inc (IL, USA). Custom designed 2′-*O*-methyl-protected short hairpin RNA (shRNA) and custom oligonucleotides were custom synthesized from Integrated DNA Technologies Inc. (IA, USA).

### Animals

Multi-timed pregnant C57BL/6 female mice (E16 on delivery) were purchased from Charles River Laboratories. All animal procedures used in this study were approved by the Vanderbilt University Institutional Animal Care and Use Committee and were performed in accordance with the Association for Research in Vision and Ophthalmology (ARVO) Statement for the Use of Animals in Ophthalmic and Vision Research and in compliance with ARRIVE guidelines.

### Design and synthesis of AS-Eng-(or NS)-shRNA–lipid conjugates

The 2′-*O*-methyl-protected short hairpin RNA (shRNA) oligonucleotides that incorporate mouse ENG mRNA specific sequence or a non-sense sequence were synthesized and purified using HPLC system according to our recently published protocol^37^. Briefly, anti-sense ENG sequences (*SEQ ID mENG seq-1, SEQ ID mENG seq-2, SEQ ID mENG seq-3* as shown bellow) were extensively BLAST searched to determine no significant overlap with any other mouse mRNA transcript. BLAST search was performed on the non-sense sequence as well to confirm non-recognition of any transcribed mouse sequence. The anti-sense ENG and the non-sense sequences are located within the loop of the hairpin structure as shown in Fig. 1. A self-complementary sequence was incorporated to form the stem of the shRNA hairpin. This sequence is largely responsible for the formation and the stability of the hairpin secondary structure. Each shRNA was computationally designed via energy minimization to achieve the formation of the hairpin structure. Each of the optimized shRNA-oligonucleotides was coupled to a Cy3 dye (fluorophore) and C6 amino group to facilitate conjugation to the diacyl-lipid. The 3′ end was coupled to a BHQ2. Finally the shRNA was conjugated to the diacyl-lipid according to our previously described method with slight modification^27^. Briefly, amine-functionalized hairpin-shaped RNA was reacted with tenfold molar excess of dibenzocyclooctyne-PEG4-N-hydroxy-succinimidyl ester (DBCO-PEG4-NHS) predissolved at 25 mM in DMSO. The reaction was carried out for overnight at room temperature at a 1 mM oligonucleotide concentration in 30% DMSO and 70% PBS with 8 mM diisopropylethylamine (DIPEA). The product was then diluted threefold in water and the excess reagents were removed by centrifugal filtration using a filter with a 3 kDa molecular weight cut-off (Amicon Ultracel 10K from Millipore, Billerica, MA) and washed twice using PBS (Life Technologies Corporation; Grand Island, NY), and then reacted with fivefold molar excess of DSPE- PEG2000-azide for 24 h at a 0.1 mM oligonucleotide concentration in 50% methanol, 50% water. The reaction was then diluted in water then purified using 10 kDa (Amicon Ultracel 10K from Millipore, Billerica, MA) molecular weight cut-off filter and washed three times using PBS. The pure conjugate was collected and diluted in PBS for characterization and in vivo applications. Molecular weight was confirmed using MALDI-TOF mass spectrometry (Voyager- DE STR Workstation) using 50 mg/mL 3-hydroxypicolinic acid in 50% water, 50% acetonitrile with 5 mg/mL ammonium citrate as a matrix or ESI-TOF MS analysis. The freshly conjugated lipid-oligonucleotides were stored at 4 °C until used. After complete synthesis, specificity and sensitivity of the purified AS-Eng and NS-shRNA–lipid conjugates were examined using complementary sequence and compared with nonsense complementary sequence as shown in supplementary Figure S3. Among several candidate sequences, mENG seq-2 was highly responsive in presence of complementary sequence and selected to use for molecular imaging of target mRNA in vivo. Both AS-Eng shRNA–lipid and NS-shRNA–lipid conjugates have similar stability profiles in serum containing medium (FBS) for at least 6 h at 37 °C (Figure S4), suggesting similar stability in vivo. In addition, the probes could hybridize to their corresponding complementary sequence as observed from increased fluorescence, suggesting their retained hairpin structures after incubation in FBS for 6 h.

The shRNA sequence of the AS-Eng shRNA is shown as:

- *SEQ ID mENG seq-1 Cy3*: 5′-mGmCmAmGmCmUmGmCmAmAmCmUmCmAmGmUmUmCmCmAmUmCmAmUmUmAmCmGmGmGmCmUmGmC-3′. mG means 2′-OMe protected G, mC means 2′-OMe protected C, mA means 2′-OMe protected A, mU means 2′-OMe protected U.
- *SEQ ID mENG seq-2 Cy3*: 5′-mGmCmAmGmCmAmCmUmGmUmGmAmUmGmUmUmGmAmCmUmCmUmUmGmGmCmGmCmUmGmC-3′.
- *SEQ ID mENG seq-3 Cy3*: 5′-mGmCmUmCmGmUmUmUmGmAmCmCmUmUmGmCmUmUmCmCmUmGmGmAmAmAmGmAmUmCmGmAmGmC-3′.
- *SEQ ID for NS sequence Cy3*: 5′-mCmCmGmGmUmUmUmAmGmUmUmCmCmUmGmUmUmCmUmGmUmUmGmUmCmUmUmCmAmCmCmGmG-3′.

Sequence positions in target mRNA:

- For ENG seq-1 Mus musculus endoglin (Eng), transcript variant 1, mRNA NM_007932.2: 756 GCCAAGAGTCAACATCACAGTGCT 779.
- For ENG seq-2 Mus musculus endoglin (Eng), transcript variant 1, mRNA NM_007932.2: 1073 CCGTAATGATGGAACTGAGTTGCA 1096.
- For ENG seq-3 Mus musculus endoglin (Eng), transcript variant 1, mRNA NM_007932.2: 1204 ATCTTTCCAGGAAGCAAGGTCAAA 1227.

### Dynamic light scattering (DLS)

DLS measurements were achieved by following a slightly modified protocol of our previously published method^24^. Briefly, on a Malvern Zetasizer Nano ZS (Malvern Instruments, Inc.; Westborough, MA). Particle measurements were performed at a concentration of 10 μM shRNA–lipid in PBS (Life Technologies Corp.; Carlsbad, CA). These measurements were performed in triplicate.

### Transmission electron microscopy (TEM)

TEM imaging was acquired^24^ by mounting the shRNA–lipid probes on 300-mesh copper grids and stained with 2% uranyl acetate. Samples were subsequently imaged on the Philips/FEI Tecnai T12 electron microscope (Hillsboro, OR) at various magnifications.

### In vivo and ex vivo imaging of mRNA in mouse OIR

To generate the OIR mouse model, dams with their pups were treated with 75% oxygen for 5 days from postnatal day 7 (P7) to P12^38^. On P12, pups were removed from the hyperoxic chamber and stayed with the nursing mother in normal air condition for additional 5 days. At P17 AS-Eng (or NS)-shRNA–lipid conjugates in sterile saline were injected intraperitoneally at a dose of 0.5 mg/kg. After 18 h, AS-Eng (or NS)-shRNA–lipid dependent fluorescence imaging was performed in vivo. Briefly, mice were anesthetized with ketamine/xylazine, eyes were dilated with 1% tropicamide, and fluorescent images were acquired using a confocal scanning-laser microscopy-imaging system (LSM 710 META Inverted, Jena, Germany). Then, ex vivo fluorescence imaging was performed to localize AS-Eng (or NS)-shRNA–lipid derived fluorescence in ocular tissues in OIR retina. After imaging, animals were sacrificed, enucleated and the globes were fixed in 10% neutral buffered formalin (NBF). Retinas were dissected and blocked/permeabilized in 10% donkey serum with 1% Triton X-100 and 0.05% Tween 20 in TBS for 2 h and were then counter-stained for IBA1 and IB4 conjugated to Alexafluor-dyes (Life Technologies; Grand Island, NY). The tissues were then mounted with Prolong Gold mounting medium with DAPI (Life Technologies; Grand Island, NY). Images were taken using an epifluorescence Nikon Eclipse T*i*-E inverted microscope (Melville, NY).

#### Macrophage depletion using intraperitoneal injection of clodrosome and imaging using intravitreal Injection of AS-Eng shRNA–lipid

Clodrosome (0.1 mL/10 g, Encapsula NanoSciences LLC.; Brentwood, TN) or PBS as control was injected intraperitoneally into P14 mouse OIR pups and in consideration of its depletion efficacy after 2 days^39^, AS-Eng shRNA–lipid was injected intravitreally (1.5 μL) using Hamilton syringes with 33 GA-19° point style custom cut needles (Hamilton Company, Reno, NV). Eighteen hours later, animals were sacrificed and retinal tissues were stained and analyzed for IBA1 and IB4, as described above.

#### Macrophage infiltration promoted by intravitreal injection of CCL2 in adult mice

Recombinant mouse CCL2/MCP-1 protein (NBP2-22772, Novus Biologicals, LLC.; Centennial, CO) 50 μg was dissolved in 500 μL sterile saline. Under anesthesia, C57BL/6 mice were anesthetized with ketamine/xylazine, and in each animal one eye was dilated with 1% tropicamide, and were intravitreally injected with 1.5 μL of the freshly prepared CCL2/MCP-1 solution using Hamilton syringes with 33 GA-19° point style custom cut needle (Hamilton Company, Reno, NV) and kept the other eye as un-injected control, followed by intraperitoneal injection of AS-Eng shRNA–lipid (0.5 mg/kg). Eighteen hours later, animals were sacrificed and retinal tissues were stained and analyzed for IBA1 and IB4, as described above.

#### Imaging of mRNA in mouse model of laser-induced choroidal neovascularization (LCNV)

To induce CNV in C57BL/6 mice, laser-induced ruptures of the Burch's membrane were performed with an 532 nm green laser photocoagulator mounted on a slit-lamp (Nidek Inc.; San Jose, CA). Four lesions were created in both the left and right eyes of each mouse. Laser parameters used were 100-μm spot size, 0.1-s duration, and 0.3 Watts. On day-3 post-laser treatment, mice were divided into two groups and received systemic injections of AS-Eng shRNA–lipid at a concentration of 0.5 mg/kg in PBS. Eighteen hours after the probe-injection, the mice were sacrificed and the eyes were fixed in neutral buffer formalin (NBF) for 30 min. The anterior segments and lenses were removed while submerging the eye in NBF solution. The choroid-Bruch’s membrane-RPE complex was dissected as previously described^40,41^ and tissues were stained and analyzed for IBA1 and IB4, as described above.

#### In vivo biodistribution of plasma half-life measurement of shRNA–lipid conjugates

For biodistribution assays, 4 to 6 weeks old adult C57BL/6 mice were used. Cy3-shRNA–lipid (without the quencher) conjugates in sterile saline were injected intraperitoneally at a dose of 0.5 mg/kg. Blood samples were collected for plasma half-life measurements from deeply anesthetized animals at 0 min, 5 min, 10 min, 0.5 h, 1 h, 2 h, and 4 h (n = 4 for each time point, duplicate experiments). Blood samples were pooled by cardiac puncture into a heparinized syringe into a 1.5 ml heparinized tube on ice, followed by removal of the heart, lung, liver, kidney, spleen, leg muscle and lymph nodes. The fluorescence intensity in the organs were quantified using Xenogen IVIS 200 fluorescence imaging system (PerkinElmer, USA) at excitation wavelength of 550 ± 5 nm and emission wavelength of 570 ± 5 nm (n = 4 animals). The blood samples were centrifuged at 6000 rpm for 5 min. Plasma samples were transferred to a clean tube and fluorescence intensities were measured using Synergy MX microplate reader (BioTek, USA) at excitation wavelength of 550 ± 5 nm and emission wavelength of 570 ± 5 nm (n = 4).

### Fluorescence in situ hybridization (FISH)

Fluorescence in situ hybridization (FISH) imaging was performed according to a previously described method^42^. Briefly, formalin-fixed paraffin-embedded (FFPE) OIR mouse eyes were sectioned in 6-µm thick slices and were deparaffinized in xylene, followed by dehydration in an ethanol series. Tissue sections were then incubated in citrate buffer (10 nmol/L, pH 6) maintained at a boiling temperature (100–103 °C) using a hot plate for 15 min, rinsed in deionized water, and immediately treated with 10 μg/mL protease plus reagent (Advanced Cell Diagnostics, Hayward, CA) at 40 °C for 30 min in a HybEZ hybridization oven (Advanced Cell Diagnostics, Hayward, CA). Hybridization with target probes, preamplifier, amplifier, and fluorescence detection using TSA Plus fluorescence detection kit (PerkinElmer, Hopkinton, MA) were performed in multistep procedures according manufactures instruction (Advanced Cell Diagnostics, Hayward, CA). Assays were performed in parallel with positive and negative controls, to ensure interpretable results. The endogenous housekeeping marker *PPIB* (Advanced Cell Diagnostics, Hayward, CA) was used as positive control to assess both tissue RNA integrity and assay procedure. The bacterial gene *DapB* (Advanced Cell Diagnostics, Hayward, CA) was used as negative control to assess background signals.

### Cell culture

MRMECs were cultured in T-75 cell culture flasks (Thermo Fisher Scientific; Wilmington, MA) coated with attachment factor (Cell Systems; Danvers, MA) and in growth medium consisting of endothelial basal medium (EBM; Lonza; Walkersville, MD) supplemented with 2% FBS (Lonza) and endothelial cell growth supplements (EGM SingleQuots; Lonza), according to our previously described method^24^. All cultures were incubated at 37 °C, 5% CO_2_ and 95% relative humidity (20.9% oxygen). The cells were cultured in 96-well plates and treated with shRNA–lipid in complete growth medium. Passages 4 to 6 were used to assess the toxicity of the imaging probes.

### Hybridization of AS-Eng shRNA–lipid

To determine target specificity, AS-Eng shRNA–lipid conjugate (1 nM in sterile PBS) was titrated with ENG-recognition complementary sequence (AS-compl) or nonsense oligo (NS-oligo) at a concentration (0.1 μM). Fluorescence intensities were measured using a microplate reader (Biotek, Winooski, VT) and plotted as a function of time. Signal to noise was determined by the fluorescence ratio of the AS-compl vs the NS-oligo. Experiments were performed at least three times with n = 3 for each experimental group.

### Cell viability assay

Cell viability assays were performed using our previously described method^24^. Briefly, an EZViable Calcein AM Fluorometric Cell Viability Assay Kit (BioVision, Milpitas, CA, USA) was used to quantify the number of viable cells. MRMECs were cultured on sterile black 96 well plates under growth conditions. At 75% confluence, MRMECs were treated with 0–0.5 µM AS-Eng shRNA–lipid in complete medium, 0 to 0.5 µM NS-shRNA–lipid in complete medium or 70% ethanol as a positive control for 8 h. After treatment, the cells were washed with cold PBS (Life Technologies Corporations). MRMECs were then exposed to a buffered (1:500) calcein AM solution and incubated at 37 °C for 30 min. Fluorometric readings were performed using a microplate reader (Biotek; Winooski, VT). Fluorescence intensity was plotted on the Y-axis and represented as % live cells. Experiments were performed at least three times with n = 3 for each experimental group.

### Imaging ENG mRNA in MRMECs using AS-Eng shRNA–lipid

Cells were plated at a seeding density of 25,000 cells per well in a pre-coated (attachment factor; Cell Systems; Danvers, MA4) 4-well glass chamber slides (Lab-tek II 154526) in complete EBM growth media (cc3129, Lonza; Walkersville, MD) supplemented with 10% FBS (Gibco) and incubated overnight at 37 °C, 5% CO_2_ and 95% relative humidity. The following morning, cells were treated with 0.5 µM of AS-Eng shRNA–lipid with or without 400 ng/mL of 2.3 mg/L PMA (phorbol 12 myristate 13 acetate, p8139-1MG, Sigma-Aldrich, St. Louis, MO) supplemented with 2% FBS in growth medium and incubated for 24 h. After treatment, cells were washed twice with DPBS (Gibco, 14190-144) and fixed in 10% NBF (EMD milipore; R04586-76) for 15 min. Cells were washed with DPBS and mounted EverBrite mounting media with DAPI (Biotium; 23002).

### Statistics

Data were expressed as mean ± % SDM and statistical differences among groups were determined by one-way analysis of variance (ANOVA) using Prism 6 (Graph- Pad, San Diego, CA) followed by Bonferroni post hoc test to determine significant differences between specific groups. A ‘p’ value < 0.05 was considered statistically significant.

## Supplementary Information

Supplementary Information.
