# Supplementary material for: A novel method for visualizing and tracking endogenous mRNA in a specific cell population in pathological neovascularization
Source: Sci Rep. 2021 Jan 28;11:2565. doi: 10.1038/s41598-021-81367-5 (PMC7844016; doi:10.1038/s41598-021-81367-5)
Supplement: Supplementary file 1 — Supplementary Information. [file 41598_2021_81367_MOESM1_ESM.pdf]

Supplementary Information

A novel method for visualizing and tracking endogenous mRNA in a specific cell population in pathological neovascularization

MD Imam Uddin,[a,b*] Tyler C. Kilburn,[a] Sara Z. Jamal,[a] Craig L. Duvall,[b] and John S. Penn,[a,c]

Authors' Affiliations

[a]Department of Ophthalmology and Visual Sciences, Vanderbilt University School of Medicine, Nashville, TN, USA.

[b]Department of Biomedical Engineering, Vanderbilt University, Nashville, TN, USA.

[c]Department of Cell and Developmental Biology, Vanderbilt University School of Medicine, Nashville, TN, USA.

Running Title: *In vivo* molecular imaging of endoglin mRNA to predict neovascularization.

A

B

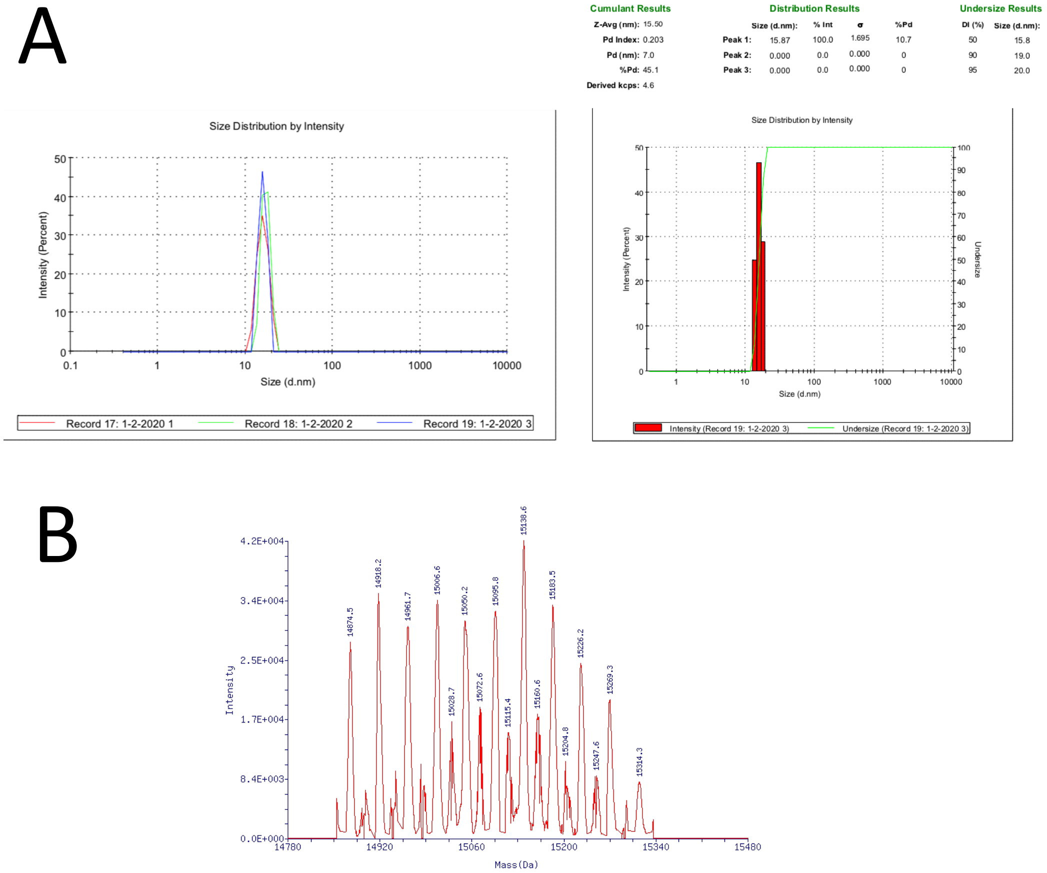

Figure S1: Size distribution and polydispersity index measurement of the shRNA-lipid conjugate in PBS using dynamic light scattering (DLS) and molecular weight measurement using ESI-TOF. (A) DLS measurements showed that, sample contains one major population by volume suggesting good measurement quality. Polydispersity index (PDI) value of 0.203 demonstrates a broad size range within the population suggesting the presence of multiple species/nanoparticles. (B) The ESI-TOF MS data for shRNA-lipid conjugates at around 15 kDa also showed a series of MS from multiple shRNA-lipid conjugates with a series of PEG lengths suggest multiple species of the conjugates that might contribute to the polydispersity.

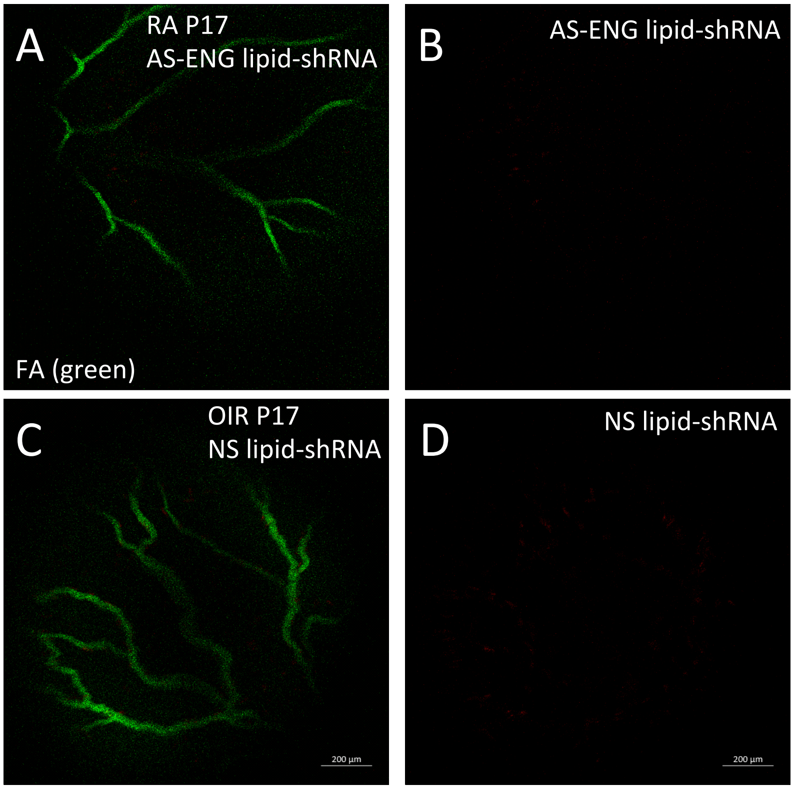

Figure S2: AS-Eng shRNA-lipid conjugates incorporating anti-sense sequence complementary to endoglin mRNA were not detectable in healthy control retinas. In addition, fluorescence was minimally detectable when a NS-shRNA-lipid probe was used in OIR retina at P17. (A-B) After intraperitoneal injections of the AS-Eng shRNA-lipid in aged-matched healthy control animals, fluorescence signals, presumably in response to probe binding, was minimally observed in healthy room air (RA) control retinas. (C-D) A nonsense-shRNA-lipid conjugate was minimally detectable in P17 OIR retinas suggesting that complementary sequence for nonsense-shRNA-lipid is not present in OIR retina and thus minimally detectable due to non-specific binding of the probe, suggesting the high specificity of AS-Eng shRNA-lipid for target sequence.

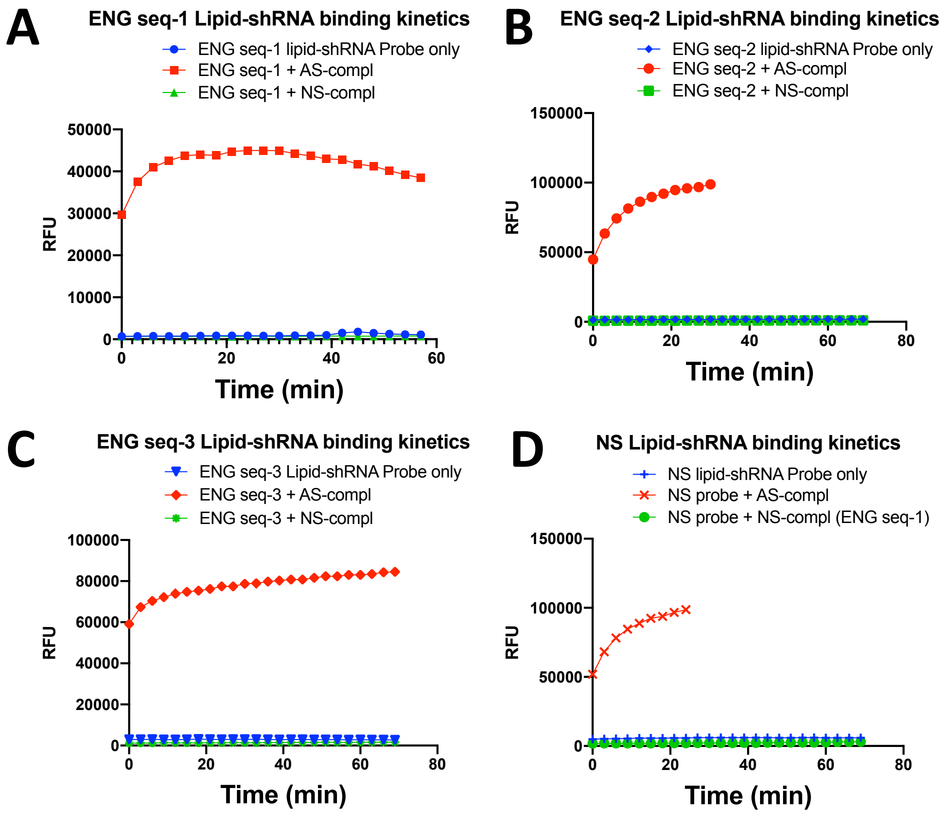

Figure S3: Sensitivity and specificity of AS-Eng shRNA-lipid conjugates. Non-overlapping sequences targeting ENG mRNA were selected using RNA secondary structure prediction program MFOLD [1] and then narrowing the best selected sequence using a second computer program, OLIGOWALK [2] which identifies probe sequence that binds most stably to their complementary sequence. A nonsense-shRNA-lipid was designed by selecting random sequence and BLAST searched to avoid nonspecific binding. Complementary sequence designed for ENG seq-1 was used monitor off-target of the nonsense-shRNA-lipid. (A-C) The 2'-MeO nucleotides protected AS-Eng shRNA-lipid conjugates were highly specific for their complementary sequences and were stable in presence of nonsense complementary sequence (NS-comp). (D) In this probe binding hybridization kinetic experiment, nonsense-shRNA-lipid also shows high-level of specificity for its perfect complementary sequence and remains non-responsive to the endoglin specific sequences, suggesting the low levels of nonspecific binding of the nonsense-shRNA-lipid to other mRNA.

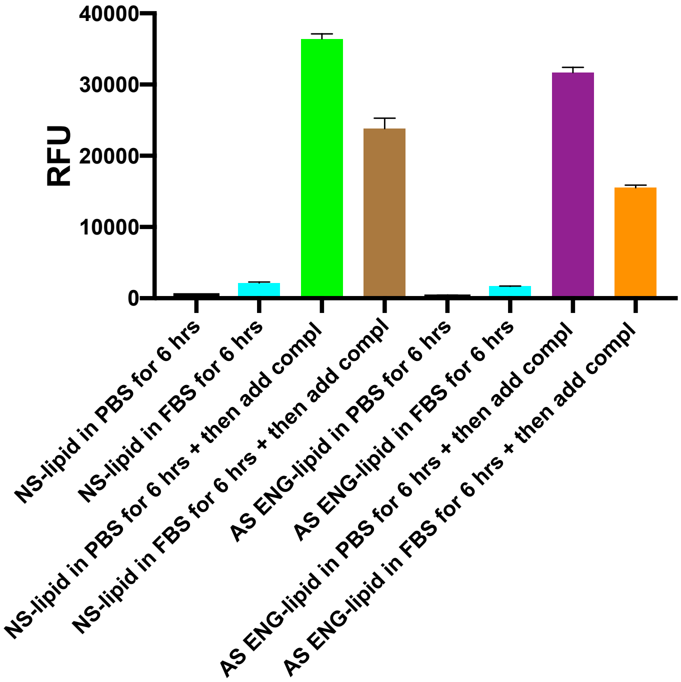

Figure S4: Comparative stability of AS-Eng shRNA-lipid and NS shRNA-lipid conjugates were monitored in serum containing medium. Probes were incubated with fetal bovine serum (FBS) or PBS for 6 hours at 37 ^{o}C. Both NS-shRNA-lipid and AS-Eng shRNA-lipid conjugate were stable in FBS as well as in PBS for at least 6 hours, suggesting similar stability *in vivo*. In addition, the probes responded to their corresponding complementary sequence after 6 hours in FBS or PBS by emitting fluorescence signals, suggesting their retained hairpin structures before hybridization to the complementary sequence.

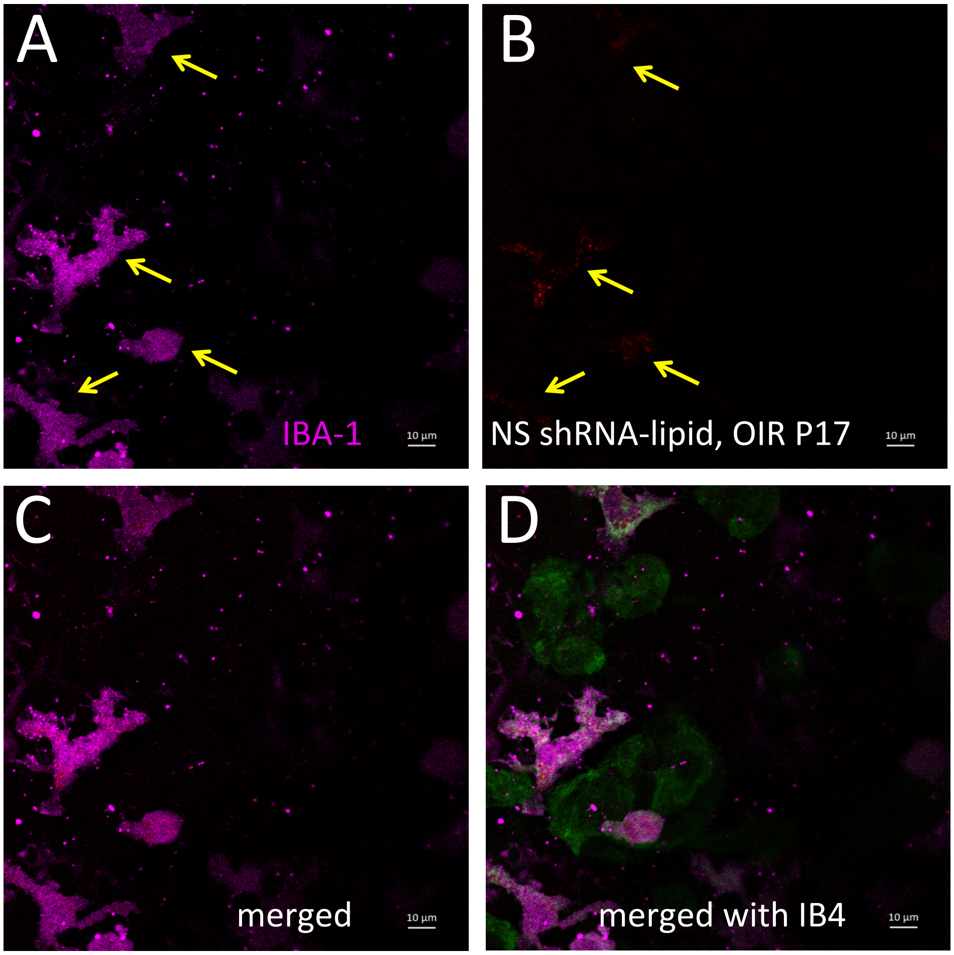

Figure S5: Sensitivity of NS-shRNA-lipid conjugates in mouse OIR retina. (A-D) When a NS-shRNA-lipid conjugate was used, minimal levels of fluorescence punctate were detected in IBA1 positive macrophages (yellow arrows) in mouse OIR at P17 (B). Nonsense sequence was designed computationally and BLAST searched to confirm no significant overlap with any mouse mRNA transcripts for nonspecific binding. Scale bar 10 µm

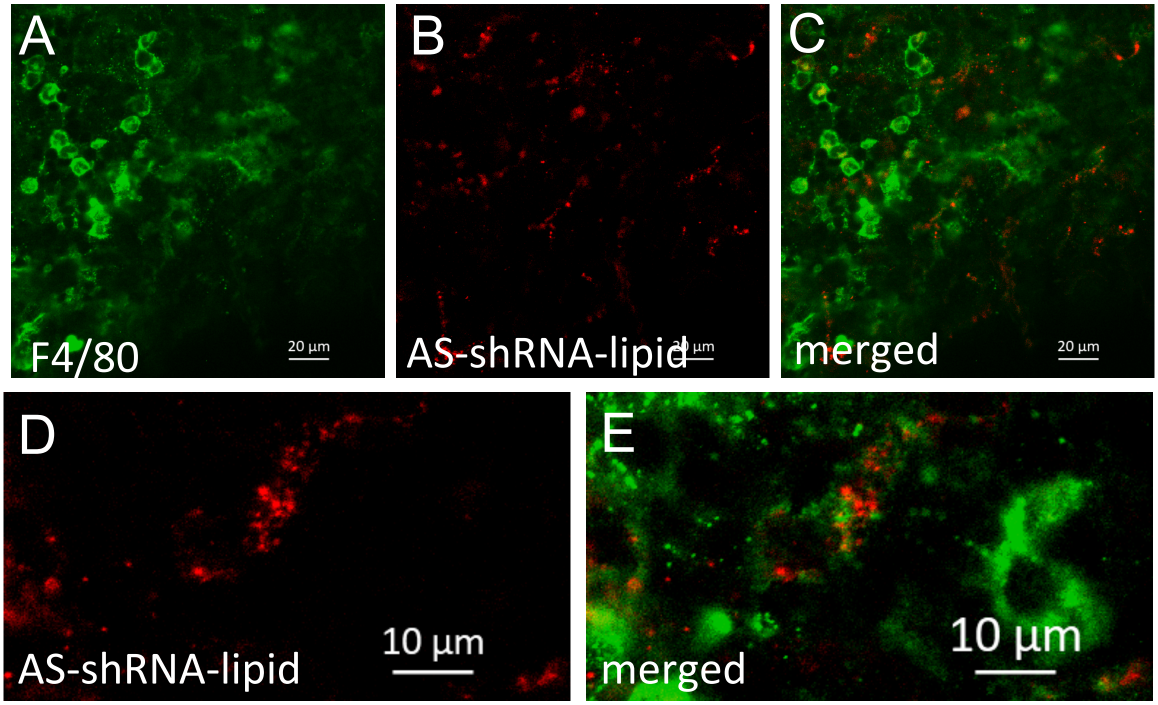

Figure S6: Co-localization of AS-Eng shRNA-lipid derived fluorescence with F4/80 positive cells in lymph nodes isolated from P17 OIR mouse. The P17 OIR mice were intraperitonelly injected with AS-Eng shRNA-lipid conjugates, eighteen hours post-injection; lymph nodes were isolated and analyzed *ex vivo*. (A) F4/80 was used to visualize the macrophages. (B) Strong fluorescence from AS-Eng shRNA-lipid is clearly visible in the lymph nodes. F4/80 positive staining was intense at the surface of the whole lymph node as observed in A; some but not all F4/80 positive cells were associated with AS-Eng shRNA-lipids. (C) A and B merged. (D-E) showing higher magnification images of the AS-Eng shRNA-lipids associated F4/80 positive cells.

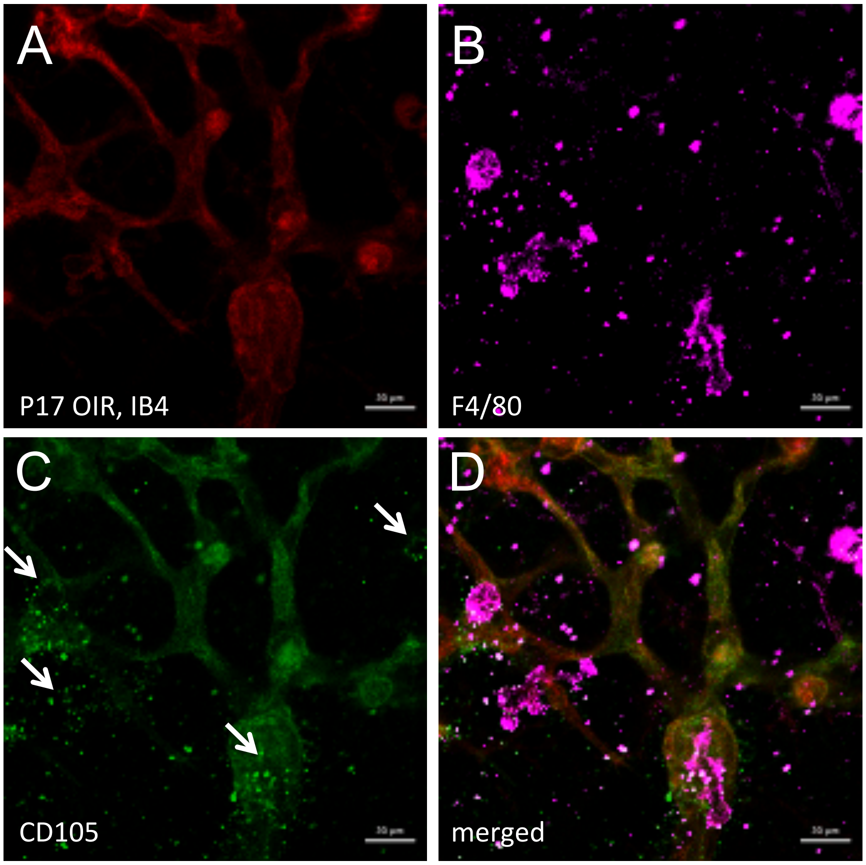

Figure S7: Endoglin (CD105) protein was co-localized with F4/80 positive cells in flat mount P17 OIR retinas using immunostaining. Isolectin B4 was used to counter stain the retinal vasculature (A), F4/80 was used to stain the macrophages (B) and CD105 protein staining (C) was performed on retinal flatmounts from P17 OIR mice. (D) A, B, C merged. Scale bar 10 μm.

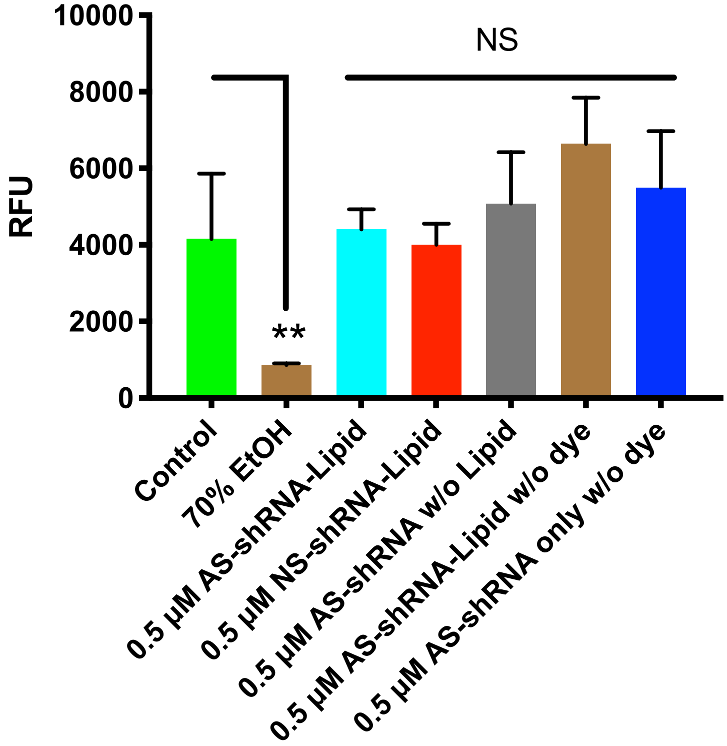

Figure S8: Cytotoxicity of shRNA-lipid was monitored using retinal microvascular endothelial cells. Live-dead assay was performed using Calcein AM. shRNA-lipid did not significantly reduce MRMEC viability at 0.5 μM concentrations compared to normal serum treated cells.

MS-data for the oligonucleotides:

Instrument:	MS-IALTQ-07	Operator ID:	3395
Acquired:	10/17/2018 10:15 PM	Reviewed:	10/17/2018 10:52 PM

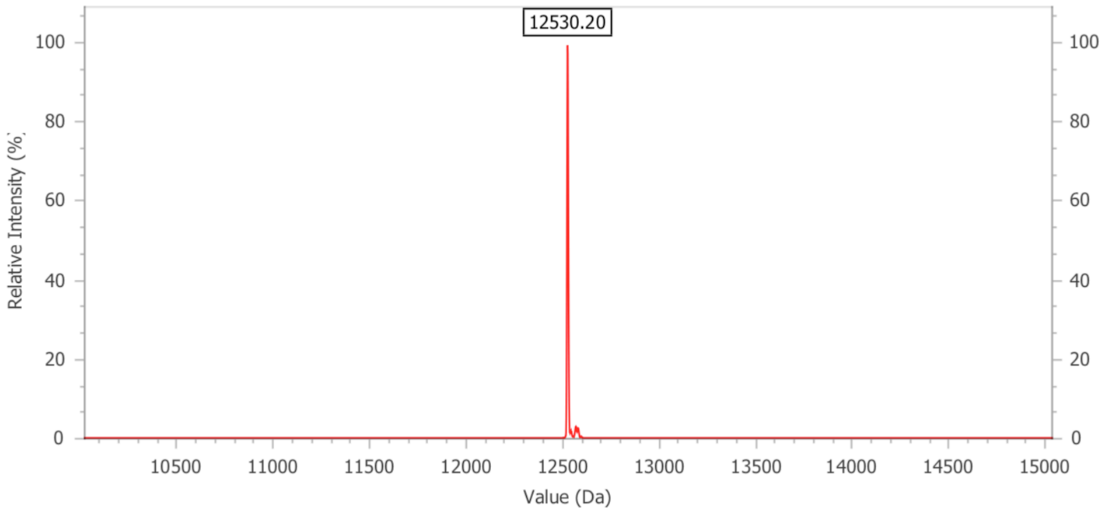

**Sequence Name:**	**MI-9-2018-mENG seq-1 Cy3**
**Sequence:**	**5'- /5AmMC6//iCy3/mGmCmA mGmCmU mGmCmA mAmCmU mCmAmG mUmUmC mCmAmU mCmAmU mUmAmC mGmGmG mCmUmG mC/3BHQ_2/ -3'**
**Calculated Molecular Weight:**	**12529.6**

Instrument: MS-IALTQ-07 Operator ID: 3395
Acquired: 10/17/2018 10:15 PM Reviewed: 10/17/2018 10:52 PM

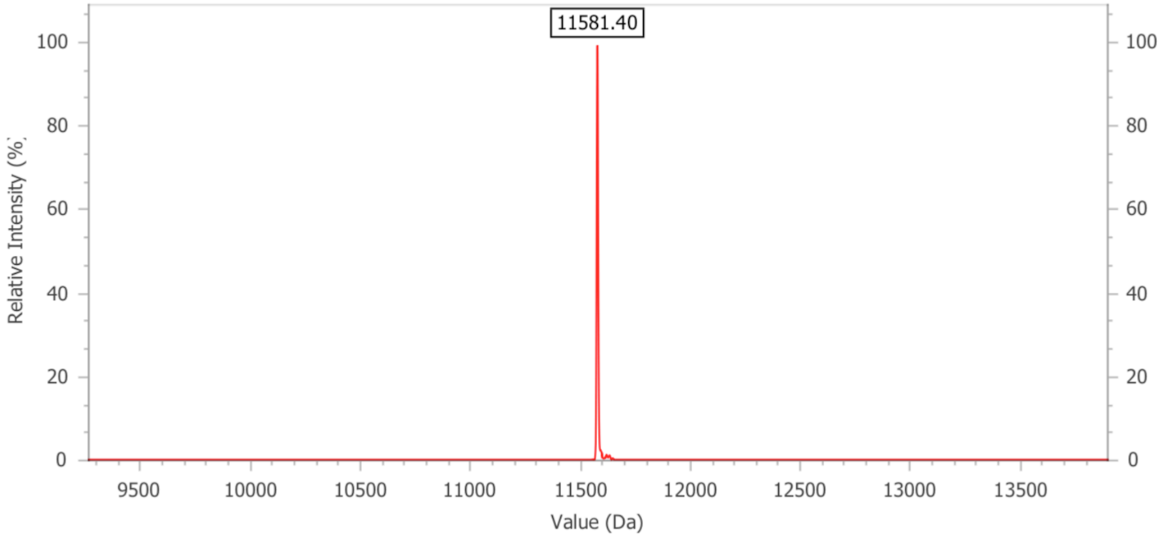

Sequence Name: MI-9-2018-mENG seq-2 Cy3

Sequence: 5'- /5AmMC6//iCy3/mGmCmA mGmCmA mCmUmG mUmGmA mUmGmU mUmGmA mCmUmC mUmUmG mGmCmG mCmUmG mC/3BHQ_2/ -3'

Calculated Molecular Weight: 11581.0

Measured Molecular Weight: 11581.40

Instrument: MS-IALTQ-02   Operator ID: 3457
Acquired: 10/18/2018 12:22 PM   Reviewed: 10/18/2018 12:37 PM

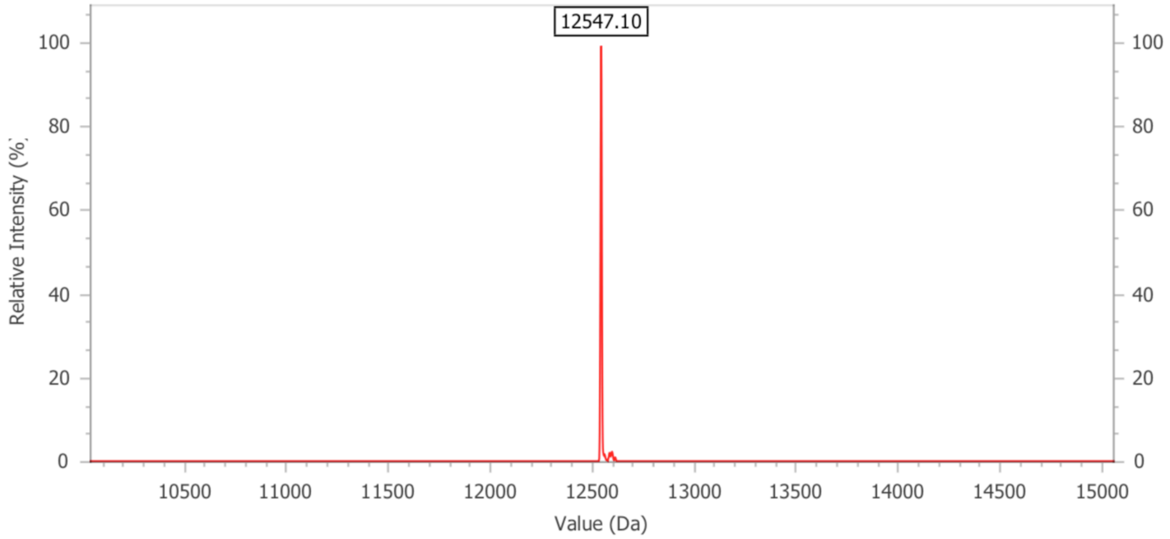

Sequence Name: **MI-9-2018-mENG seq-3 Cy3**

Sequence: **5'- /5AmMC6//iCy3/mGmCmU mCmGmU mUmUmG mAmCmC mUmUmG mCmUmU mCmCmU mGmGmA mAmAmG mAmUmC mGmAmG mC/3BHQ_2/ -3'**

Calculated Molecular Weight: **12547.6**

Measured Molecular Weight: **12547.10**

Instrument: MS-IALTQ-02
Acquired: 10/18/2018 12:22 PM

Operator ID: 3457
Reviewed: 10/18/2018 12:37 PM

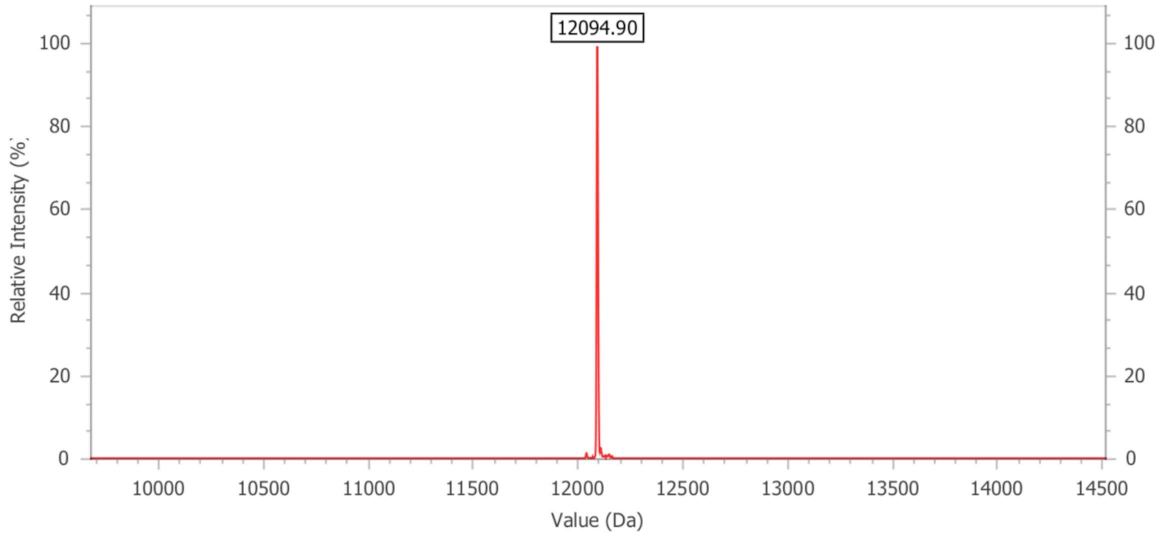

Sequence Name: **MI-9-2018-NSense Cy3**

Sequence: **5'- /5AmMC6//iCy3/mCmCmG mGmUmU mUmAmG mUmUmC mCmUmG mUmUmC mUmGmU mUmGmU mCmUmU mCmAmC mCmGmG /3BHQ_2/ -3'**

Calculated Molecular Weight: **12096.2**

Measured Molecular Weight: **12094.90**

Instrument: MS-IALTQ-11 Operator ID: 3455
Acquired: 10/21/2018 10:22 PM Reviewed: 10/21/2018 10:30 PM

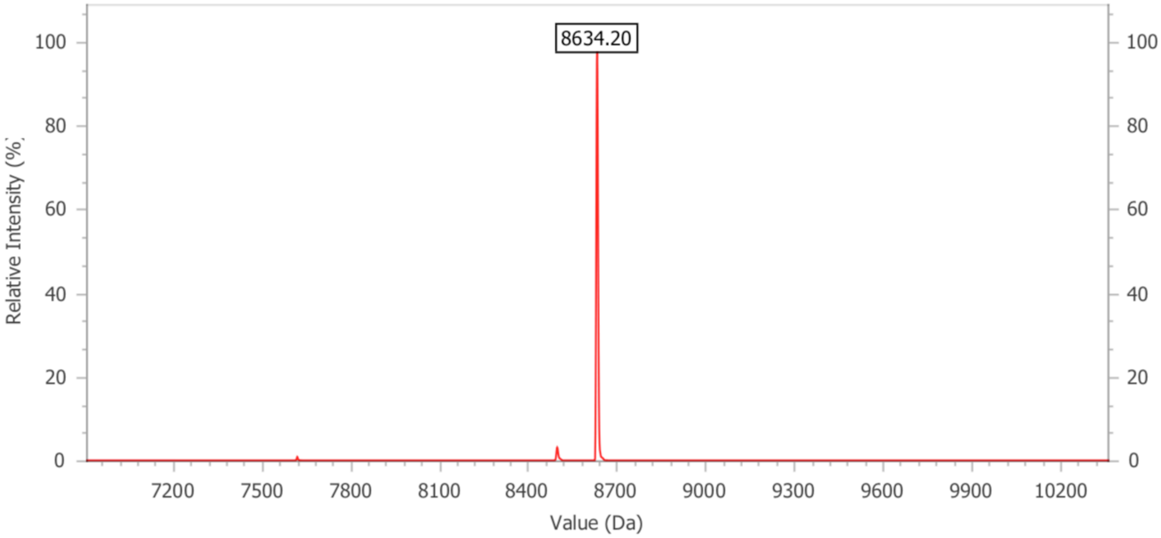

Sequence Name: **MI_9-2018 mENG seq-1 comp**

Sequence: **5'- TTC CGT AAT GAT GGA ACT GAG TTG CAT T -3'**

Calculated Molecular Weight: **8633.7**

Measured Molecular Weight: **8634.20**

Instrument: MS-IALTQ-11 Operator ID: 3114
Acquired: 10/15/2018 6:35 PM Reviewed: 10/15/2018 7:09 PM

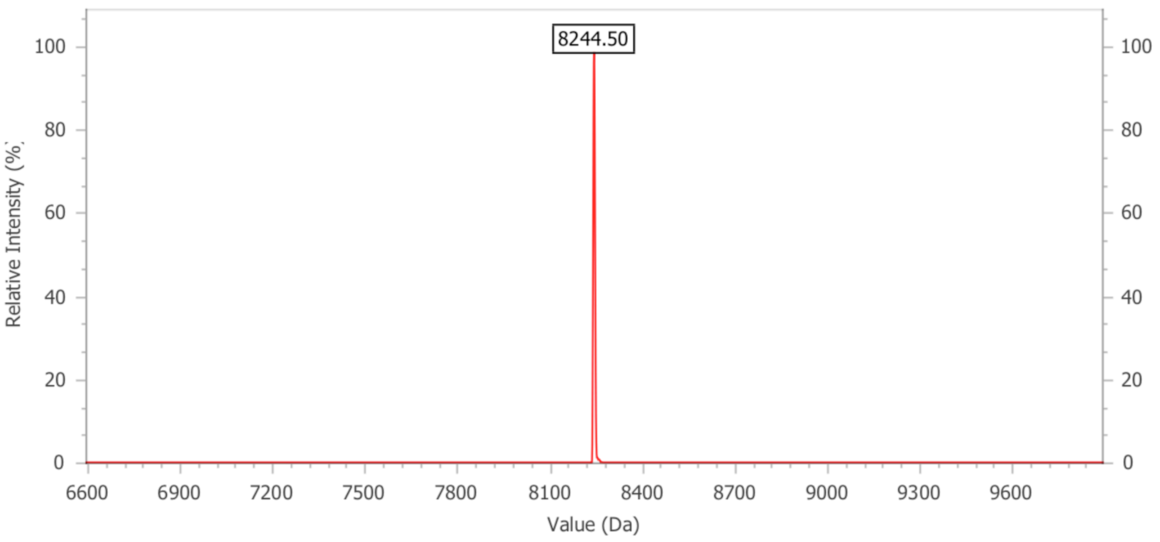

Sequence Name: **MI_9-2018-mENG seq-2 Compl**

Sequence: **5'- TTG CCA AGA GTC AAC ATC ACA GTG CTT -3'**

Calculated Molecular Weight: **8243.4**

Measured Molecular Weight: **8244.50**

Instrument:	MS-IALTQ-11	Operator ID:	3114
Acquired:	10/15/2018 6:35 PM	Reviewed:	10/15/2018 7:09 PM

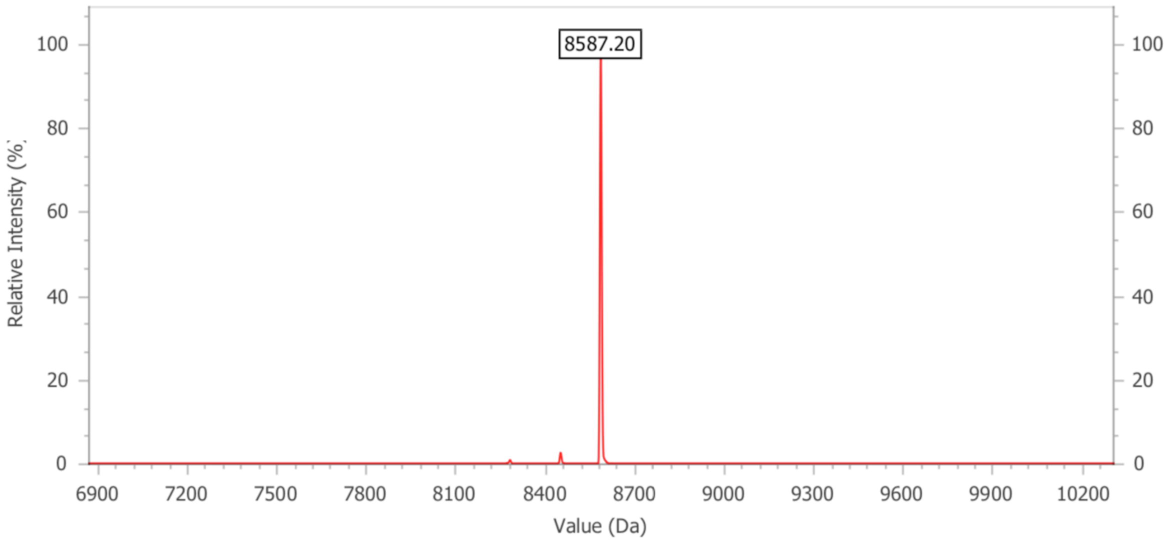

Sequence Name: **MI_9-2018-mENG seq-3 Compl**

Sequence: **5'- TTA TCT TTC CAG GAA GCA AGG TCA AAT T -3'**

Calculated Molecular Weight: **8586.6**

Measured Molecular Weight: **8587.20**

Instrument: MS-IALTQ-12 Operator ID: 4082
Acquired: 10/15/2018 7:09 PM Reviewed: 10/15/2018 7:19 PM

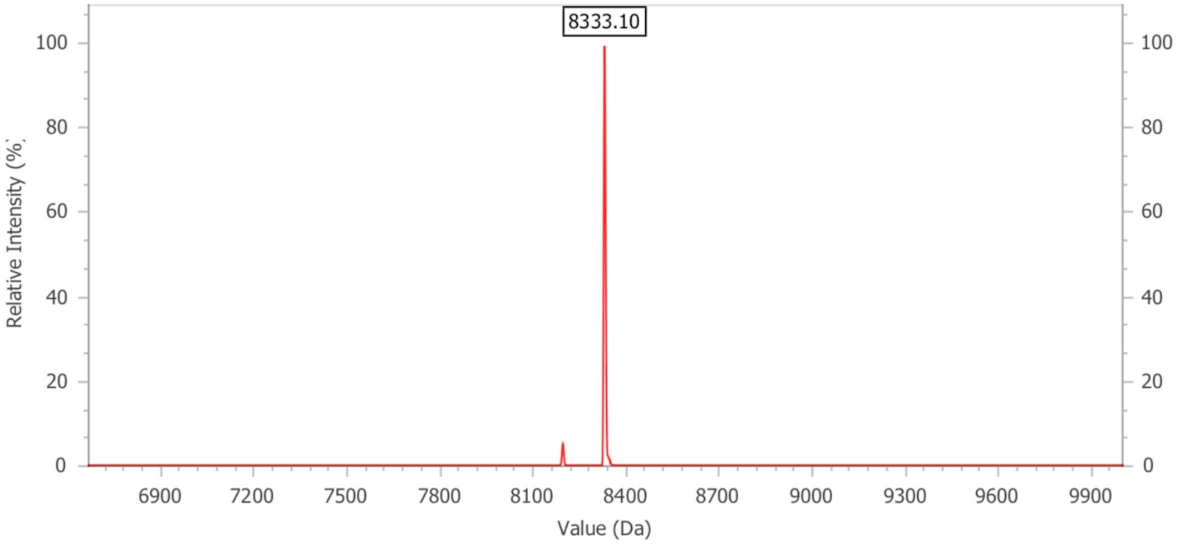

Sequence Name: **MI_9-2018 NS compl**

Sequence: **5'- TTG AAG ACA ACA GAA CAG GAA CTA ATT -3'**

Calculated Molecular Weight: **8333.5**

Measured Molecular Weight: **8333.10**

Instrument: MS-IALTQ-12
Acquired: 10/15/2018 7:08 PM
Operator ID: 4082
Reviewed: 10/15/2018 7:19 PM

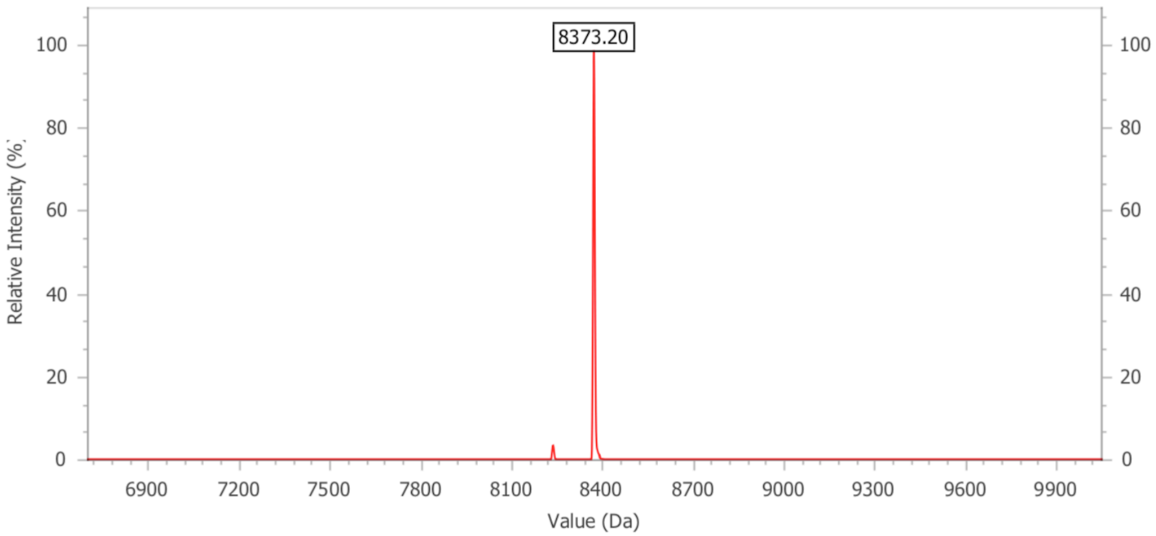

Sequence Name: **MI_2018 NS SMM compl**

Sequence: **5'- TTG AAG ACA ACA GAA GAG GAA CTA ATT -3'**

Calculated Molecular Weight: **8373.5**

Measured Molecular Weight: **8373.20**

MS-data for the oligonucleotides without BHQ to use for biodistribution assays:

Instrument:	MS-IALTQ-05	Operator ID:	2212
Acquired:	1/14/2019 11:00 AM	Reviewed:	1/14/2019 11:24 AM

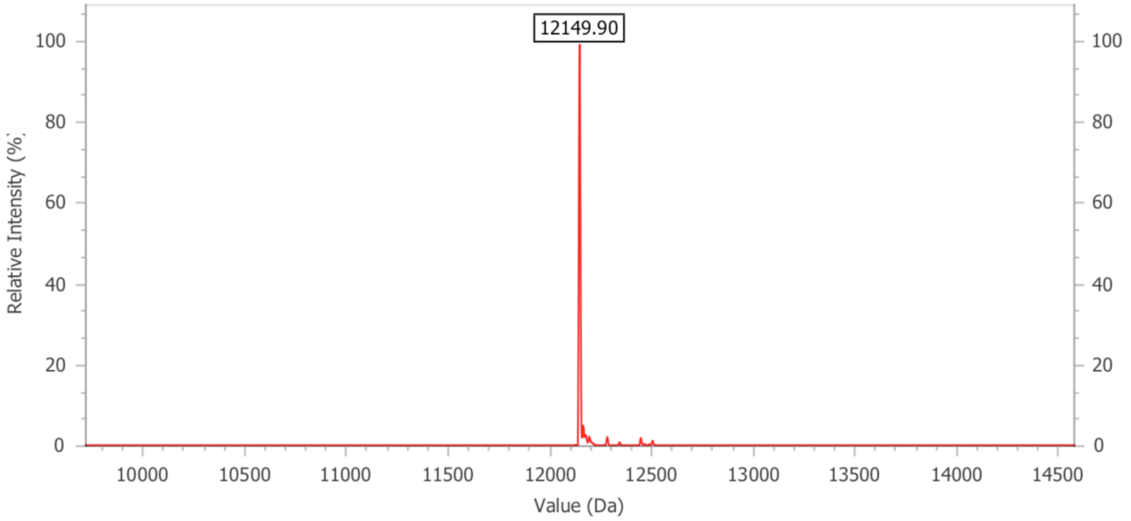

**Sequence Name:**	**MI-12-2018-mENG Cy5 Thiol**
**Sequence:**	**5'- /5ThioMC6-D//iCy5/mGmCmA mGmCmU mGmCmA mAmCmU mCmAmG mUmUmC mCmAmU mCmAmU mUmAmC mGmGmG mCmUmG mC -3'**
**Calculated Molecular Weight:**	**12148.5**
**Measured Molecular Weight:**	**12149.90**

Instrument: MS-IALTQ-09 | Operator ID: 3533999
Acquired: 1/18/2019 2:10 PM | Reviewed: 1/18/2019 3:05 PM

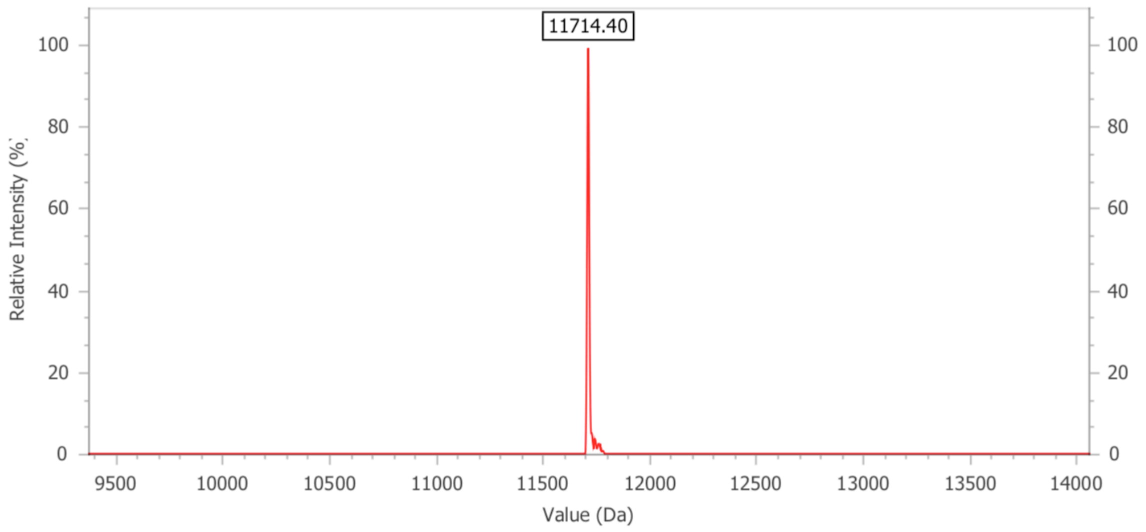

Sequence Name: **MI-12-2018-NSense Cy5 Thiol**

Sequence: **5'- /5ThioMC6-D//iCy5/mCmCmG mGmUmU mUmAmG mUmUmC mCmUmG mUmUmC mUmGmU mUmGmU mCmUmU mCmAmC mCmGmG -3'**

Calculated Molecular Weight: **11715.0**

Measured Molecular Weight: **11714.40**

HPLC data for the purified compounds

Analytical RP HPLC Report

Sample ID: 204222679-32 PHG A4 118160-H14
Instrument: HPLC #14 (Offline) Operator: HTA
Acquired: 1/18/2019 6:10:30 PM Reviewed: 1/18/2019 9:14:36 PM

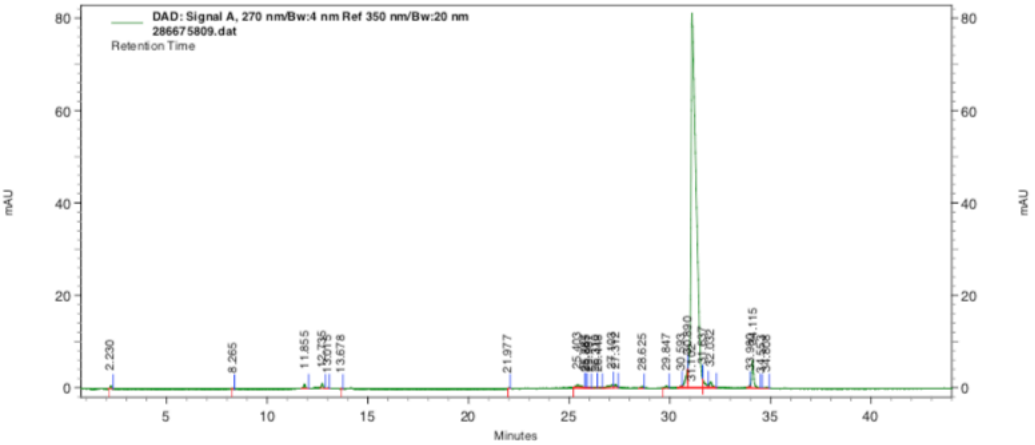

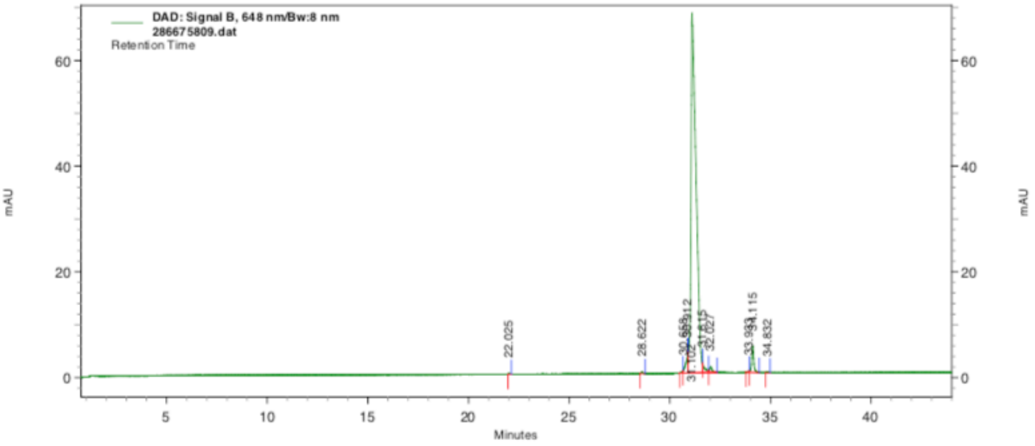

ESI-TOF data for shRNA-lipid conjugates.

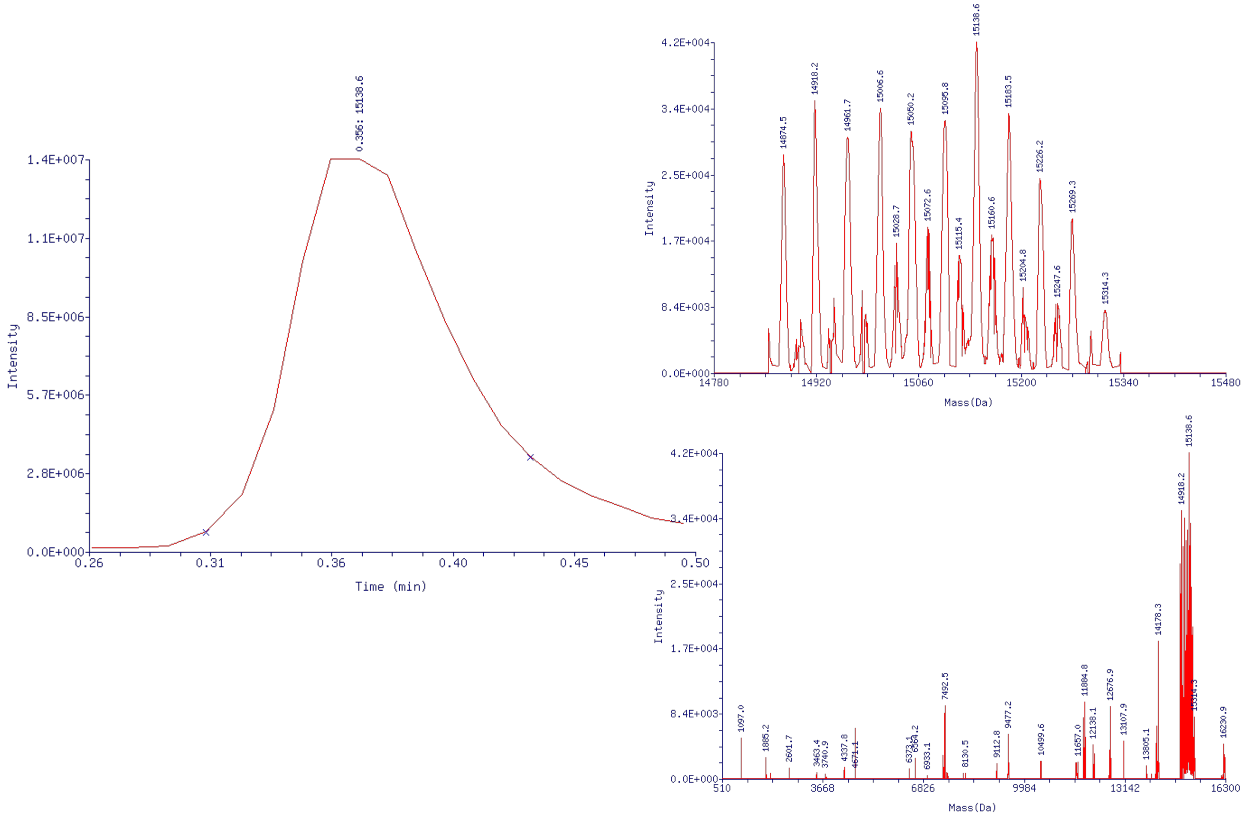

MS around 15.1 kDa with several PEG lengths as differentiated by MS-difference of 44 Da.

References

1. Mathews, D.H., Sabina, J., Zuker, M. & Turner, D.H. Expanded sequence dependence of thermodynamic parameters improves prediction of RNA secondary structure. *J Mol Biol* **288**, 911-940 (1999).
2. Mathews, D.H., Burkard, M.E., Freier, S.M., Wyatt, J.R. & Turner, D.H. Predicting oligonucleotide affinity to nucleic acid targets. *RNA* **5**, 1458-1469 (1999).
